# The mechanical properties of *Arabidopsis thaliana* roots adapt dynamically during development and to stress

**DOI:** 10.1126/sciadv.aeb0032

**Published:** 2026-02-18

**Authors:** Luis Alonso Baez, Astrid Bjørkøy, Francesco Saffioti, Sara Morghen, Dhika Amanda, Michaela Tichá, Maarten Besten, Anastasiia Ivanova, Joris Sprakel, Bjørn Torger Stokke, Thorsten Hamann

**Affiliations:** ^1^ Department of Biology, Norwegian University of Science and Technology, Trondheim 7491, Norway.; ^2^ Department of Physics, Norwegian University of Science and Technology, Trondheim 7491, Norway.; ^3^ Laboratory of Biochemistry, Wageningen University and Research, Wageningen, Netherlands.

## Abstract

Mechanical properties of plant cells and tissues change dynamically, influencing plant growth, development, and interactions with the environment. Despite their central roles in plant life, current knowledge of how these properties change in vivo is very limited. Here, we have combined Brillouin microscopy and molecular rotors to investigate stiffness, viscosity, and porosity in living *Arabidopsis thaliana* seedling roots during differentiation and in response to stress and genetic manipulation. We found that mechanical properties change in a cell- and tissue-specific manner. The properties change dynamically during differentiation to support directional cell expansion. Cell type–specific adaptation of the properties is induced within hours in response to stress or changes in cell wall metabolism. Hyperosmotic stress–induced reduction of cell wall stiffness requires intact abscisic acid metabolism and cell wall integrity signaling. The findings form the foundation for future studies to characterize the regulatory mechanisms linking cell wall homeostasis, signaling, and mechanical properties in plants.

## Teaser

Brillouin microscopy reveals in vivo dynamics of mechanical properties during plant development and response to stress.

## INTRODUCTION

The mechanical properties of plant structures, ranging from the subcellular to the organ level, are key determinants for plant growth, development, and interactions with the environment (*1*–*4*). These properties arise from the interplay between turgor pressure, molecular crowding, and subcellular structures such as the cytoskeleton, cell wall, or nuclear envelope (*5*–*7*). Their dynamic nature is tightly linked to biological function, yet our understanding of how mechanical properties vary across tissues and scales remains limited (*8*–*10*). In particular, subepidermal plant cell layers have been characterized predominantly through indirect methods while in vivo measurements have largely been restricted to surface tissues or whole organ analyses (*11*–*15*).

Recently, noninvasive technologies have become available enabling in vivo analyses of subepidermal mechanical properties with subcellular and temporal resolution. Two complementary approaches exemplify these advances: Brillouin microscopy and 4,4-difluoro-4-bora-3a,4a-diazas-indacene (BODIPY)–based molecular rotors (*16*–*18*). Molecular rotors report on local mechanical properties through environmentally driven changes in rotor configuration, which affect its fluorescence lifetime. In this study, we used CarboTag dyes to assess hydrodynamic porosity (mesh size), reflecting the space available for probe rotation within the cell wall matrix (*19*). For instance, in cellulose-rich environments with larger pores, the probe will have more freedom to rotate, resulting in lower lifetime values. In contrast, in pectin-rich environments with smaller pores, probe rotation is hindered and slowed down, resulting in higher lifetime values (*19*). Brillouin microscopy provides label-free measurements of intrinsic material mechanical properties by detecting frequency shifts and changes in the linewidth of the inelastically scattered light from the sample, with increases in frequency shift or linewidth implying increased stiffness and viscosity (*20*). Stiffness is related to material deformation or compression, while viscosity is a resistance to flow or movement between adjacent layers. Brillouin microscopy uses light to detect the effect of spontaneous, thermally induced density fluctuations in a material. These fluctuations transiently compress the material and produce local volume changes. The Brillouin frequency shift reports how strongly the material resists these thermally driven volume changes (longitudinal modulus). The Brillouin linewidth reflects how quickly these fluctuations lose coherence, reflecting viscous or dissipative processes (longitudinal viscosity) (*21*, *22*). The calculation of the longitudinal modulus and longitudinal viscosity requires that the refractive index and mass density of the material are known. Brillouin microscopy, unlike Young’s modulus measurements, which produce values in the MPa range, captures mechanical behavior at GHz frequencies, yielding higher moduli in the GPa range. Stiffness and viscosity changes are not necessarily correlated, highlighting the value of measuring both parameters to dissect complex mechanical properties (*22*, *23*).

Simultaneous analysis of multiple mechanical properties is essential to fully understand the molecular processes controlling them in plant tissues and cell types during development and environmental interactions. This approach opens previously unexplored avenues to elucidate causal relationships between mechanical patterns observed at the subcellular level with those detected at tissue and organ levels (*8*, *10*, *24*). Here, we begin to provide these insights by generating in vivo maps of the mechanical properties of *Arabidopsis thaliana* (Arabidopsis) root layers along developmental axes and examining how the properties change during differentiation with single-cell resolution, in response to stress and to genetic alterations in cell wall metabolism and signaling. These results establish a framework to investigate the root mechanical properties across spatial scales, developmental stages and environmental interactions, laying the groundwork for uncovering regulatory mechanisms and developing mechanobiological in vivo models (*25*–*28*).

## RESULTS

### Tissue-specific mechanical maps of Arabidopsis root tips

Tissues in Arabidopsis seedling roots are organized as radially concentric cylindrical layers (epidermis, cortex, endodermis, and stele). Because of their different functions in development and stress responses, it has been postulated that individual tissue layers have distinct mechanical properties (*29*, *30*). However, this has never been experimentally validated in vivo in a noninvasive manner because direct in vivo measurements of mechanical properties in subepidermal tissues have been technically challenging and mostly relied on indirect observations (*4*, *10*, *11*, *13*).

**Fig. 1.**
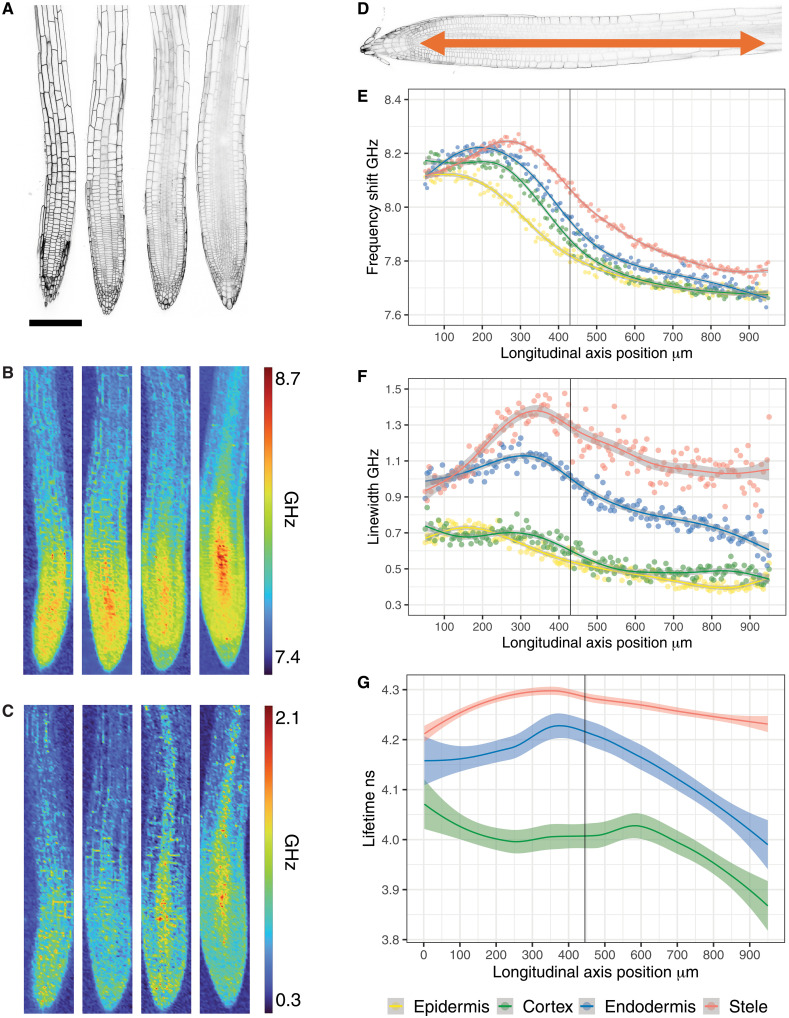
Maps of the mechanical properties of the Arabidopsis root tip. [(A) and (B)] Left to right, image of epidermis, cortex, endodermis, and stele planes in a seedling root tip. (**A**) Confocal images using p35S::LTI6b-GFP plasma membrane marker. Scale bar, 150 μm. (**B**) Frequency shift and (**C**) linewidth heatmaps, 150 by 1000 μm, of the areas scanned in (A). (**D**) Direction of quantification performed along the longitudinal root axis. (**E**) Frequency shift, (**F**) linewidth and (**G**) cell wall porosity quantification. The vertical line indicates the average position of the transition zone identified with respect to the cortex file. *n* = 9 to 12 roots for each tissue layer for Brillouin data. *n* = 10 roots for molecular rotors data.

To close this major knowledge gap, we characterized the four major tissue types in 6-day-old Arabidopsis seedling roots, using confocal and Brillouin microscopy. We treat the stele here as a major tissue type because we cannot separate developing xylem and phloem cells. First, we obtained fluorescence images of Col-0 roots expressing the p35S::LTI6b-GFP plasma membrane marker (Fig. 1A). Subsequently, we scanned the same areas to establish the local mechanical properties (Fig. 1, B and C). For each tissue, we quantified the spatial profile of the frequency shift (related to stiffness) and the linewidth (related to viscosity) along the developmental axis of the root, from the meristem zone above the stem cell niche to the end of the elongation zone (Fig. 1D). The stele plane showed a unique frequency shift profile (Fig. 1E). While its values in the vicinity of the stem cell niche were comparable to those of the other tissues, the frequency shift increased markedly in the central meristem. It remained elevated relative to the surrounding tissues before decreasing just before the transition zone and dropping further in the elongation zone. Frequency shift levels in the epidermis, cortex, and endodermis layers exhibited similar values in the meristematic region. The positions of the maximum frequency shifts differed between the tissues. The maximum frequency shift was located near the stem cell niche in the epidermis, in the middle region of the meristem for both cortex and endodermis and a few cells before the transition zone in the stele (defined as the position in the cortex file where cells start to elongate) (*31*). The epidermis values gradually declined toward the elongation zone, whereas cortex and endodermis values increased slightly in the meristem before declining. Among the three tissues, the epidermis consistently exhibited the lowest frequency shift values, while cortex and endodermis remained higher. Linewidth measurements in the stele were markedly higher than in the other three tissues along the developmental axis (Fig. 1F). The lowest values were observed near the stem cell niche, followed by a gradual increase that peaked in the central meristem before decreasing toward the elongation zone. In the endodermis, linewidth values began at levels comparable to the stele near the stem cell niche but exhibited only a modest increase in the meristem, followed by a decrease toward the transition and elongation zones. The cortex and epidermis showed similar patterns, with consistently lower linewidths than the stele and endodermis. Although the changes were more subtle, both displayed a gradual decline from the meristem to the elongation zone.

To validate these findings, we visualized local changes in cell wall porosity using CarboTag-BDP molecular rotors (*19*). Median plane profiles of roots revealed elevated fluorescence lifetimes in the meristem zone relative to the elongation zone (fig. S1A). We quantified the longitudinal porosity profile of cell walls along the developmental axis for distinct root tissues. The epidermal layer was not quantified because of chemical quenching of the probe at the root surface (*19*) (Fig. 1G). In both stele and endodermis layers, lifetime values increased from the stem cell niche to the end of the meristem zone and then declined to lower values in the elongation zone. The lifetime values in the cortex layer were higher in the stem cell niche, decreased to a plateau in the meristem zone, and decreased further in the elongation zone. These results are consistent with our Brillouin-based measurements and confirm that mechanical properties change dynamically along the longitudinal root axis.

Our measurements identified distinct and tissue-specific profiles associated with stiffness and viscosity in the seedling root in vivo. The patterns suggest that cells undergo dynamic changes in their mechanical properties during the developmental transition from division to elongation, a process that has been reported to take between 1 (*32*, *33*) and 3 days (*34*). Stiffness, viscosity and porosity exhibit similarities along the developmental axes but also show differences, highlighting the importance of investigating more than one mechanical property simultaneously. These observations imply the existence of regulatory mechanisms modulating mechanical properties with single-cell resolution.

### Mechanical properties of single cells

**Fig. 2.**
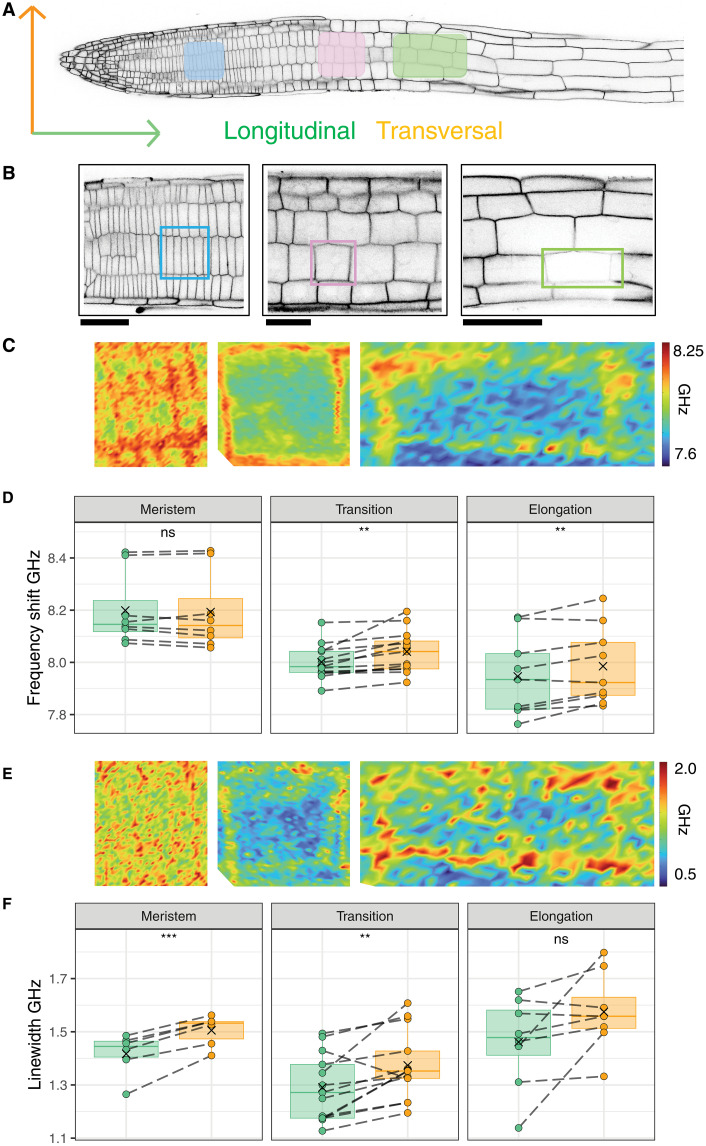
Anisotropic mechanical properties of Arabidopsis cell walls in the cortex layer. (**A**) Orientations of cell walls and confocal images of the cortex plane visualized with p35S::LTI6b-GFP. Regions where imaging was performed are indicated for the meristem (blue), transition (pink), and elongation zone (green). (**B**) Confocal images of the meristem (left), transition zone (middle), and elongation (right) zones, used to locate the cortex planes to generate the following heatmaps. (**C**) Heatmaps of the frequency shift in cells in the meristem (50 by 40 μm, left), transition (32 by 32 μm, middle), and elongation zone (30 by 60 μm, right). (**D**) Quantification of the frequency shifts at cell walls. (**E**) Heatmaps of the linewidth in cells at the meristem (left), transition zone (middle) and elongation zone (right). (**F**) Quantification linewidth at cell walls. *n* = 8 to 10 different roots for each zone. A paired *t* test was used to obtain the statistics for individual zones. *P* values are reported as ns > 0.05, **P < 0.01, ***P < 0.001.

As differentiation progresses, anisotropic distribution of mechanical properties between transversal and longitudinal cell walls is thought to be modified in a tightly controlled manner to allow cell expansion along the longitudinal axis (*14*, *18*, *35*). We chose the cortex layer as a model to investigate in vivo the temporal and spatial dynamics of mechanical properties in individual cells along the developmental axis (Fig. 2A). We established and compared mechanical properties of longitudinal and transversal cell walls in the cortex cell layer in three different developmental stages (Fig. 2, B to F). In the meristematic zone, frequency shifts were similar in both longitudinal and transversal walls, while linewidth was higher in the transversal walls. In the transition zone, both frequency shift and linewidth values were higher in transversal walls compared to longitudinal ones. In the elongation zone, frequency shifts in transversal cell walls were increased compared to longitudinal cell walls, while no significant differences in the linewidth existed between them. The frequency shift values of the cortex cell walls decreased from the meristem to the elongation zone, in agreement with the results of the global map of the root tip. Notably, the decrease was more pronounced in the longitudinal walls compared to the transversal walls, which is consistent with the direction of cell elongation along the developmental axis. We also detected heterogeneous mechanical properties in individual cell walls with nonuniform frequency shift and linewidth values, suggesting local variation in mechanical properties possibly reflecting locations where fresh cell wall materials are being synthesized and/or deposited (transition and elongation zones; Fig. 2, C and F). In complementary experiments using CarboTag-BDP, transverse cell walls exhibited higher porosity than longitudinal walls across all developmental stages examined (fig. S1B). Next, we repeated the experiments with mutant seedlings that have reduced cellulose levels [*procuste1/cellulosesynthaseA6* (*prc1-1*), approximately 70% reduction] and exhibit profound changes in xyloglucan oligomers and cell wall organization [*xylosyltransferase1 xylosyltransferase2* (*xxt1 xxt2*)] or defects in pectin biosynthesis, cellulose organization, and cell-cell adhesion [*quasimodo2* (*qua2*)] in seedling roots (fig. S2) (*36*–*39*). Viscosity was not anisotropic anymore in the three genotypes examined (fig. S2C). In *xxt1 xxt2* and *qua2* cortex cells anisotropy with respect to stiffness is also not detectable but it is still present in *prc1-1*. To summarize, these results suggest that mechanical anisotropy may already be present in the meristem zone because viscosity and porosity seemed notably higher in transverse cell walls while stiffness differences were not significant (Fig. 2, D and F, and fig. S1B). This would be consistent with expansion being limited while cell division activity is ongoing (*40*). In the transition and elongation zones, longitudinal cell walls were less stiff than transversal cell walls, allowing directional elongation as predicted by theoretical models and previous experiments (*14*, *24*, *35*). The results from the analysis of the cell wall mutants suggest that changes in cell wall composition and structure have differential effects on mechanical properties. Anisotropy within a cell may be maintained for one type of mechanical property while simultaneously it is lost for another one.

To investigate spatial variation in stiffness and viscosity within an individual cell further, we selected initially root hair cells, since they form a well-characterized and representative model for polarized cell expansion (*41*, *42*) (fig. S3). The p35S::LTI6b-GFP reporter was used as an indicator for plasma membrane integrity and root hairs, ranging in lengths from 30 to 65 μm, were analyzed (fig. S3, B and C). In 5 of 19 cells we detected an ellipsoidal region with elevated frequency shift and linewidth, probably representing the nucleus (fig. S3D). We decided to test this hypothesis using seedlings expressing a p35s::NLS-GFP construct, which labels nuclei (fig. S4). We observed in the nuclei of hypocotyl and root epidermal cells increased frequency shift and linewidth compared to the surrounding cytoplasm (fig. S4, A to C). We performed also a more detailed analysis of root hairs by quantifying the frequency shift and linewidth in three regions, tip, middle, and base, excluding the nuclear region when present (fig. S3A). The frequency shift was significantly higher at the base compared to the middle and tip, whereas the linewidth did not differ between the three regions, suggesting that viscosity is conserved while stiffness differs between the observed areas (fig. S3E). In parallel, we measured the refractive index (*n*) of the root hair cell peripheries using quantitative phase imaging (QPI; fig. S3F), obtaining values consistent with previous reports (average *n* = 1.38) (*43*). On the basis of these measurements, we calculated the corresponding mass density (see Materials and Methods) (*44*) and, found that the longitudinal modulus at the root hair periphery ranged from 2.62 to 3.07 GPa, values typical of soft biological materials (*45*). These values exceed the megapascal range reported by atomic force microscopy, due to higher frequency operation of Brillouin microscopy (*46*). These results provide insights into the in vivo mechanical properties of subcellular structures in root and hypocotyl cells as well as root hairs. They reveal that stiffness and viscosity of nuclei and cytoplasm differ from each other in vivo in different cell types. The measurements also suggest that mechanical properties can change within a single root hair cell on the length scale of 10 to 20 μm, consistent with the observations we made in cortex cells.

### Mechanical properties differ between tissue layers in the elongation zone

**Fig. 3.**
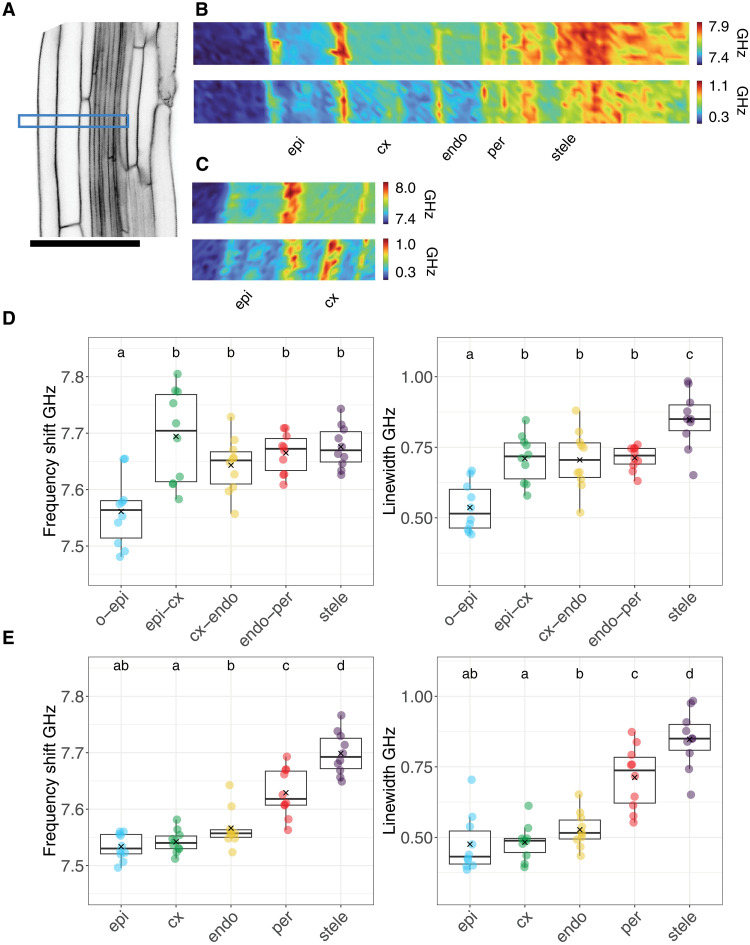
Mechanical properties of cell walls and cytoplasm in the elongation zone. (**A**) Confocal image of the median plane of a root elongation zone expressing p35S::LTI6b-GFP. The yellow rectangle indicates the area scanned with the Brillouin microscope. (**B**) Heatmaps (10 by 100 μm) of frequency shift (top) and linewidth (bottom). (**C**) Focused heatmaps (10 by 42 μm) of epidermis and cortex cells showing frequency shift (upper) and linewidth (lower). (**D**) Quantification of the frequency shift (left) and linewidth (right) of cell walls. (**E**) Quantification of the frequency shift (left) and linewidth (right) of the cytoplasm. *n* = 10 roots for each tissue layer. A repeated-measures ANOVA with Holm correction was used for statistical analysis. epi, epidermis; cx, cortex; endo, endodermis; per, pericycle.

Root cells stop dividing and start their directional growth in the elongation zone, marking a fundamental switch in differentiation state. This developmental transition is particularly sensitive to perturbations, leading to alterations in the morphology of the cell wall or plasma membrane occurring in this region first. To investigate the impact of perturbations on mechanical properties in individual cell types, we first characterized the mechanical properties of cell walls and cytoplasm in the various tissue layers in the middle part of the elongation zone under control conditions (Fig. 3, A to C). We found that frequency shifts in the outer epidermal cell walls were lower than those of all other tissues (Fig. 3D). The cell wall separating epidermis from cortex showed the highest mean frequency shift value of all cell walls examined. The remaining inner cell walls also exhibited shifts higher than the outer epidermal cell wall. The linewidth changed in a stepwise manner, with the lowest values exhibited also by the outer-epidermal wall, intermediate values in the epidermis-cortex, cortex-endodermis, and endodermis-pericycle walls and the highest values detected in the stele. In both epidermis and cortex cells, we regularly observed cytoplasmic areas with increased linewidth values compared to the remainder of the respective cell. The structures are at least 10 μm in length (Fig. 3C). In the cytoplasm, frequency shift and linewidth changed in a similar manner (Fig. 3E). Lowest values were observed in the epidermis with a gradual increase toward inner tissues and a peak in the stele. These observations suggest pronounced differences in the mechanical properties of outer epidermal versus inner cell walls and a more gradual change in the properties of the cytoplasm toward the stele.

We also examined 4-day-old seedlings to determine whether mechanical properties change with developmental age. Frequency shifts and linewidths in both cell walls and cytoplasm displayed qualitatively similar trends as observed in older seedlings. Outer tissues layers exhibited the lowest and the stele the highest values (fig. S5, A to D), suggesting that mechanical properties of the central elongation zone are stable in this time window.

### Perturbations in cell wall biosynthesis and integrity affect mechanical properties

**Fig. 4.**
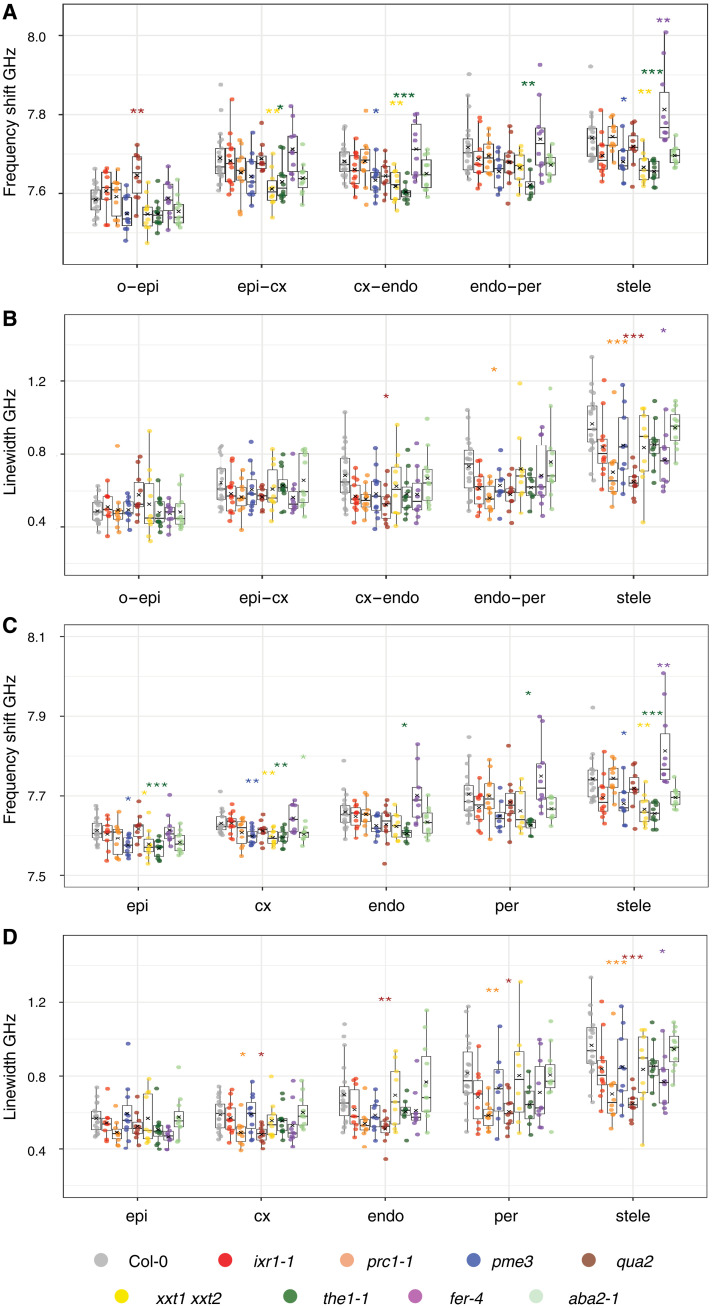
Mechanical properties of mutants affecting cell wall metabolism, CWI signaling, or ABA biosynthesis. Quantification of the mechanical properties in the main root tissues of selected mutants. (**A**) Frequency shift and (**B**) linewidth of cell walls. (**C**) Frequency shift and (**D**) linewidth of cytoplasm. *n*_Col-0_ = 19 roots and *n*_mutants_ = 9 to 11 roots. A one-way ANOVA with Dunnet’s post hoc test was used to obtain the statistics. o, outside; epi, epidermis; cx, cortex; endo, endodermis; per, pericycle. *P* values are reported as *P < 0.05, **P < 0.01, ***P < 0.001.

We examined next if genetic manipulation of cell wall metabolism and cell wall integrity (CWI) signaling affect the mechanical properties of the elongation zone in a specific manner using the following mutants: *prc1-1*; *isoxaben resistant1* (*ixr1-1*), containing a point mutation in *CELLULOSE SYNTHASE3* leading to 30% less crystalline cellulose (*47*); *pectin methylesterase3* (*pme3*), impaired in demethylesterification of homogalacturonan and affecting mechanical strength of cell walls (*48*); *xxt1xxt2* (*37*); and *qua2* (*39*). In addition, we investigated *theseus1* (*the1-1*) and *feronia* (*fer-4*) seedlings, which have defects in CWI signaling and responses to mechanical stimuli (*38*, *49*–*51*). We also included *abscisic acid deficient2* (*aba2-1*) seedlings, which produce less abscisic acid (ABA), a hormone required to regulate turgor pressure in response to hyperosmotic stress (*51*). Brillouin measurements revealed tissue-specific alterations in mechanical properties of the cell walls and cytoplasm in certain mutants (Fig. 4 and fig. S6, A to C). Compared to the corresponding cell walls in Col-0, *qua2* exhibited elevated frequency shifts in the outer epidermal cell wall; *xxt1xxt2* and *the1-1* exhibited lower frequency shifts in epidermis-cortex walls; *xxt1xxt2*, *the1-1*, and *pme3* in cortex-endodermis walls; and *the1-1* in endodermis-pericycle walls while *pme3*, *xxt1xxt2*, and *the1-1* exhibited changes in the stele walls (Fig. 4A). *fer-4* exhibited an elevated frequency shift only in the stele walls, suggesting limited impact on mechanical properties. Linewidth values were reduced in cortex-endodermis walls for *qua2*, endodermis-pericycle walls of *prc1-1*, and in the stele walls for *prc1-1*, *qua2* and *fer-4* (Fig. 4B). In the cytoplasm, reduced frequency shift values were detected in the epidermis in *pme3*, *xxt1xxt2*, and *the1-1*; in the cortex in *pme3*, *xxt1xxt2*, *the1-1*, and *aba2-1*; in the endodermis and pericycle in *the1-1*; and in the stele in *pme3*, *xxt1xxt2*, and *the1-1*, compared to Col-0 controls (Fig. 4C). Similar to corresponding cell walls, *fer-4* seedlings showed increased cytoplasmic frequency shifts in the stele. Cytoplasmic linewidths were reduced in the cortex and pericycle in *prc1-1* and in *prc1-1* and *fer-4* in the stele, compared to Col-0 (Fig. 4D). The only cell layer not exhibiting a reduced cytoplasmic linewidth in *qua2* seedlings was the epidermis.

Unexpectedly, these results suggest that impaired cellulose crystallinity or biosynthesis alone apparently do not have a major impact on stiffness. However, cellulose biosynthesis seems to be necessary for maintaining viscosity levels in certain tissues. Perturbation of THE1 (and, to a lesser extent, FER) had more pronounced effects on the mechanical properties than mutations affecting particular cell wall biosynthesis genes. These receptor kinases might participate in multiple signaling pathways in a context-dependent manner, leading to broader mechanical effects when their function is disrupted. The increased frequency shift and decreased linewidth in *fer-4* roots may be explained by a low hydration state of its cell walls, consistent with a mechanical transition from a liquid-like to a solid-like state (*23*). The results for *the1-1*, *pme3*, and *xxt1xxt2* suggest that the mechanical properties of the cortex cell layer are particularly sensitive to defects in pectin modification, xyloglucan biosynthesis, and CWI maintenance. Intriguingly cytoplasmic and wall stiffness of *the1-1*, *pme3*, and *xxt1xxt2* cortex cells is also reduced implying that changes in mechanical properties of cortex cell walls lead to adaptive changes in the cytoplasm. *qua2* seedling roots are intriguing because they only exhibit pronounced frequency shifts in the outer epidermis wall, while linewidth results indicate effects on viscosity comparable to *xxt1 xxt2*, where frequency shift changes are detectable in several cell layers. While the expression patterns of the respective genes are quite broad, the mutant seedlings exhibited distinct tissue-specific effects on mechanical properties. This implies that tissue layers have unique mechanical cell wall and/or cytoplasmic properties and that the encoded molecular components have tissue-specific functions, which are not necessarily regulated on the transcriptional level.

### Mechanical properties in the elongation zone change rapidly in response to hyperosmotic stress

**Fig. 5.**
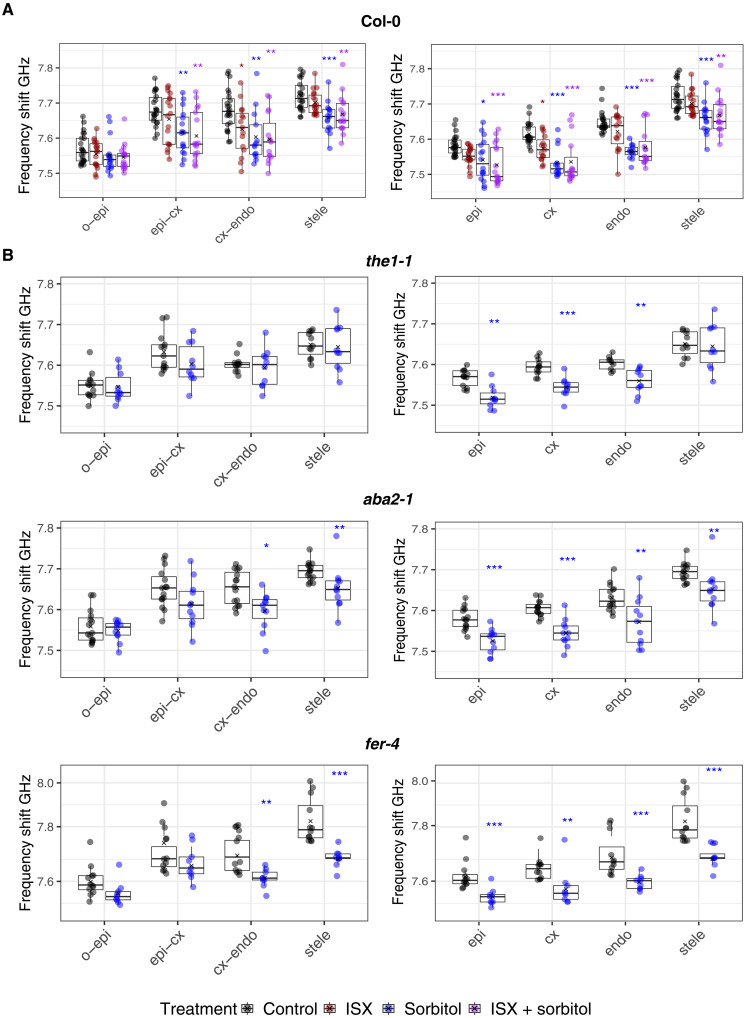
Frequency shifts in the elongation zone in response to stress. Quantification of the frequency shift in cell walls (left column) and cytoplasm (right column) in (**A**) Col-0 and (**B**) selected mutants. *n*_Col-0_, 15 to 23 roots and *n*_mutants_, 9 to 12 roots. A one-way ANOVA with Dunnet’s post hoc test was used to obtain the statistics. ISX, isoxaben. o, outside; epi, epidermis; cx, cortex; endo, endodermis. *P* values are reported as *P < 0.05, **P < 0.01, ***P < 0.001.

Previously, we showed that impairing CWI with isoxaben (ISX; a cellulose biosynthesis inhibitor) and turgor pressure reduction by sorbitol leads to decreased frequency shifts after 6 hours in Arabidopsis roots compared to controls (*51*). We did not observe differences between the combined treatment (ISX + sorbitol) and controls, suggesting that the individual effects were neutralizing each other. However, these studies lacked the spatial resolution necessary to detect tissue-specific changes in mechanical properties. Here, we investigated with higher resolution the mechanical properties of individual tissue layers in roots after treatment for 3 hours (Fig. 5 and figs. S7 to S9), when the effects on cell morphology start to be visible and sorbitol-induced ABA production is at its maximum in our model system (*51*, *52*). The outer epidermal wall did not exhibit frequency shift changes in response to any treatment (Fig. 5A). The epidermis-cortex and stele walls showed decreased frequency shifts in response to sorbitol and ISX + sorbitol treatments, while cortex-endodermis cell walls exhibited reduced frequency shifts in all treatments. In the cytoplasm, sorbitol and ISX + sorbitol treatments significantly reduced the frequency shift in all cell layers, whereas ISX alone only produced a reduction in the cortex. Linewidth values of cell walls and cytoplasm remained unchanged in response to all treatments (fig. S8A). Next, we investigated how manipulating CWI signaling and ABA biosynthesis affects the response to sorbitol. We treated *the1-1*, *fer-4*, and *aba2* seedlings for 3 hours with sorbitol and compared frequency shift and linewidth to controls (Fig. 5B and fig. S8B). We detected no differences with respect to linewidth in *the1-1*, *fer-4* between mock and sorbitol-treated samples both in cell walls and cytoplasm, like the results for Col-0 (fig. S8, A and B). In *aba2*, differences existed for linewidth in sorbitol-treated stele and epidermal cell cytoplasm as well as stele walls. *the1-1* and *fer-4* roots exhibited frequency shift reductions in the cytoplasm similar to Col-0 in response to sorbitol (Fig. 5B). However, we detected no reduction in frequency shifts in cell walls of sorbitol-treated *the1-1* seedlings. *aba2-1* and *fer-4* exhibited reduced frequency shifts in cortex-endodermis and stele walls but lacked them in outer and epidermis-cortex cell walls. Since frequency shifts were observed both in sorbitol-treated cell walls and cytoplasm while linewidths did not change, stiffness and viscosity respond apparently differently to hyperosmotic stress. *ABA2*, *THE1*, and *FER* are required for the sorbitol-induced changes in cell wall stiffness but not the cytoplasm. While *THE1* seems to be required in all cell types examined, *FER* and *ABA2* apparently have more specific activities.

## DISCUSSION

We have generated a high-resolution in vivo map of mechanical properties of the Arabidopsis seedling root tip using Brillouin microscopy to quantify frequency shift and linewidth (as proxies for stiffness and viscosity) and complemented this with porosity measurements using BODIPY-based molecular rotors. We found that the mechanical properties of different cell layers change along their respective developmental axes, highlighting how properties can change in a short period of time in a highly dynamic manner. Intriguingly, the outer epidermal cell wall exhibited consistently lower frequency shifts than internal cell walls, implying that it is not the stiffest cell wall in the root tip (*53*). Our results suggest that the mechanical properties of the root may be regulated in a context dependent manner to accommodate specific requirements arising during growth and development (*10*). Once, the different cell types are fully differentiated only the stele continues to exhibit pronounced differences. The stele actually displayed already during differentiation the highest stiffness and viscosity, likely reflecting compressive forces exerted from surrounding tissues (*54*).

We observed that stiffness changes in cortex cell walls during differentiation to support directional cell elongation in a manner previously postulated (*8*). Viscosity and porosity changed in a similar but not identical manner in the differentiating cortex cells. Our in vivo studies detected also local variability in mechanical properties of cell walls surrounding root hairs and individual cortex cells during differentiation. These observations reinforce the notion that the mechanical properties of cell walls are not always uniform and that mechanical properties can be tuned by specific cellular processes (*55*, *56*). The differences observed in the mechanical properties are probably determined by presence or absence of different cell wall components and how the ones in place are crosslinked. It is interesting to note in this context that reduction in cellulose alone (exemplified by *prc1-1*) did not have the most profound effect on stiffness but rather mutations, which either affect simultaneously several components (*xxt1 xxt2*) or CWI signaling (*the1-1*). These results suggest that either cellulose levels are here not sufficiently reduced to have a profound effect on stiffness or that even though cellulose is the load-bearing element, its loss can be complemented, to some extent, by cell wall plasticity. However, cell wall plasticity can only compensate up to a certain point for impairment of cell walls and seems to require intact CWI signaling (*57*, *58*).

Targeted genetic manipulation of cell wall metabolism (specifically xyloglucan and pectin methylesterification but not cellulose) or CWI signaling induced tissue-specific changes of mechanical properties in the elongation zone exemplified by cortex cells. Intriguingly, we observed in *xxt1 xxt2* simultaneously changes in stiffness of cortex cell walls and cytoplasm but not viscosity, while in *qua2*, we detected changes in viscosity of cell walls and cytoplasm while stiffness was only affected in the epidermis. Seedling roots exposed to hyperosmotic stress for 3 hours exhibited changes in stiffness but not viscosity. These results imply that stiffness is modified in a coordinated (cell wall–cytoplasm) manner on the subcellular level if cell wall homeostasis is disturbed while viscosity is modulated differently. The phenotypes observed in sorbitol-treated *the1-1*, *fer-4*, and *aba2-1* seedlings suggest that the modifications are mediated by CWI signaling and require changes in ABA levels. One possible scenario explaining the phenotypes observed involves a feedback loop involving CWI monitoring and ABA. Hyperosmotic stress induces changes in ABA levels modifying turgor, which in turn leads to modifications in cell wall composition/structure affecting cell wall stiffness. The CWI maintenance mechanism detects the changed cell wall stiffness and initiates adaptation involving cell wall and cellular metabolism. Loss of ABA biosynthesis (*aba2-1*) or CWI signaling (*the1-1* and *fer-4*) would “disrupt” the feedback loop at different points and prevent reduction in wall stiffness in response to hyperosmotic stress (*51*, *59*).

The results we present here illustrate how it is now possible to dissect the mechanical behavior of living plant cells and tissues in a noninvasive and systematic manner using Brillouin microscopy (*60*). By modifying cell wall homeostasis in a targeted manner and investigating the consequences on mechanical properties in vivo with subcellular resolution, we will be able to characterize the mechanisms coordinating changes in mechanical properties, biochemistry, and cell biology active during plant growth, development, and adaptation to a changing environment.

## MATERIALS AND METHODS

### Plant material

*A. thaliana* seeds of Col-0 accession were obtained from the European Arabidopsis Stock Center in Nottingham, from laboratories previously publishing them or from collaborators (table S1). Mutant alleles were confirmed via polymerase chain reaction using the Phire Plant Direct PCR Master Mix Kit (Thermo Fisher Scientific F160L). Subsequently, gel electrophoresis or Sanger sequencing (Eurofins Genomics) was performed to confirm the genotype of the plants.

### Plant growth

Seeds were surface sterilized in three steps: washed in 70% ethanol, then 10 min in 25% (v/v) bleach (Klorin), and then washed them three times with sterile water. Sterile seeds were sown on solid media plates containing 0.5× Murashige and Skoog basal medium (Merck, M5519-50L), 0.05% MES sodium salt (Merck, M3058-1KG), 1% agar (Merck, A1296-1KG), pH 5.75, and stratified 48 hours at 4°C. For Brillouin microscopy experiments, the plates were supplemented with 1% sucrose (Merck, S7903-5KG). For fluorescence lifetime imaging microscopy (FLIM) experiments, the plates did not contain sucrose. Plants were transferred to a growth chamber and grown under long-day conditions (16-hour light/8-hour dark, 150 μmol⋅m^−2^⋅s^−1^ photon flux density, 21°C, and 40% humidity) for 6 days except for the early developmental studies where plants were taken from the growth chamber after 4 days.

### Plant treatments

Six-day-old seedlings were transferred to plastic dishes (Thermo Fisher Scientific, Sterilin, 10655821) with control or treatment solutions for 3 hours. Immediately after, seedlings were mounted on microscopy slides for imaging. Control samples were treated with 0.5× Murashige and Skoog basal medium and 0.05% MES sodium salt, pH 5.75. Treatments were done separately with 600 nM ISX (Merck, 75772-50MG), 300 μM sorbitol (d-sorbitol; Merck, 56021-5KG), or a combined treatment with both compounds.

### Confocal microscopy

Fluorescent and bright-field images were obtained with an inverted SP8 Leica confocal microscope. Samples were illuminated using a 40× water-immersion objective with a numerical aperture of (NA) 1.1 (Leica). A 488-nm laser was used to excite the reporters p35S::LTI6b-GFP (localized to the plasma membrane) and p35S::NLS-GFP (localized to the nucleus) both in Col-0 background and fluorescence was collected at 500 to 550 nm using a hybrid detector and a pinhole of 1 airy unit. Bright-field images were collected simultaneously, and the same illumination parameters were used for the nonfluorescent samples. To identify the cortex cell layer for the anisotropy measurement in mutants and the cell shape for the nuclei measurement in the epidermis, we used 10 μM fluorescein diacetate. Excitation was performed as described above.

### Fluorescence lifetime imaging microscopy

Seedlings were stained with 20 μM CarboTag-BDP (*19*) probe for 1 hour and then transferred to the mounting media. Roots were examined using a Leica SP8 SMD/MP confocal microscope (Leica Microsystems) equipped with a 25× NA 0.95 water immersion objective. White-light laser 488 nm was used as an excitation line and fluorescence was captured between 500 and 550 nm. The signal was collected using a single-photon avalanche photodiode with a gain of 200%. The output was connected to a time-correlated single-photon counting (TCSPC) module PicoHarp 300 (Picoquant, Germany) and photons were captured over a period of 136 s at a repetition rate of 20 MHz. FLIM analysis was performed using SymphoTime 64 (PicoQuant, Germany), using an *n*-exponential tailfit fitting model and with the model parameter set to *n* = 2. The time range (*x* axis) in the generated TCSPC graph was adjusted to start from 0.5 ns after the instrument response function and end 1 ns before the tail. Subsequently, lifetime maps, representing the mean fluorescence lifetime per pixel in nanoseconds, were generated by adjusting the false color scale according to the lifetime histogram. In addition, the MosaicJ plugin in ImageJ was used to stitch and generate the image of the whole root (*61*, *62*).

### Quantitative phase imaging

An inverted Axio Observer Z1 microscope (ZEISS) equipped with a QPI/spatial light interference microscope module was used to determine the refractive index of root hair peripheries. A refractive index of 1.33 was set for water as a reference. Temperature in the room was maintained at 20°C for stability during measurements. A 10× objective with NA 0.3 (EC Plan-Neofluar) was used to illuminate the sample and the CELLVISTA (SLIM edition PhiOptics) software was used to control image acquisition with a Hamamatsu Orca Flash version 2 sCMOS camera. The refractive index was obtained from 17 root hairs from different roots.

### Brillouin microscopy

#### Instrumentation

Brillouin microscopy was performed using a custom-built confocal Brillouin microscopy (fig. S10A) based on a two-stage virtually imaged phase array (VIPA) configuration, following the design described in (*63*). Excitation was provided using a 532-nm laser (Cobolt, Hübner Photonics). The laser was guided through a half-wave plate, a polarized and a beam expander to a polarized beam splitter. Laser light was further directed to quarter-wave plate before entering the back port of an inverted Leica SP8 confocal microscope. Samples were illuminated using a 40× water-immersion objective NA 1.1 (Leica) and the scattered light was collected using a backscattered geometry (180°). Scattered light passed again through the quarter-wave plate and polarized beam splitter before being directed to a 4× NA 0.10 objective (Olympus) which focused the light into a fiber coupler connected to a single mode optical fiber (Thorlabs). The spectrometer consisted of two-stage VIPA etalons (OP-6721-3371-2, 500 to 600 nm, 30 GHz free spectral range, Light Machinery), vertical and horizontal physical masks to block the elastically scattered light (Raileigh peaks) and a Lyot stop (SF) (*64*), providing a spectral contrast of 75 to 80 dB (*63*). An ORCA-Quest qCMOS camera (C15550-20UP, Hamamatsu Photonics) was used to record the Brillouin spectra from the second and third light mode. The microscopy room was maintained at 20°C and the laser was mounted on a heated plate set to 37°C (according to manufacturer recommendations). During Brillouin spectra recording, the confocal microscope stage movement and image acquisition was controlled using the HCImage software (Hamamatsu Photonics). To check for perturbations and phototoxicity, samples were inspected by transmitted-light wide-field illumination before and after the Brillouin spectra were recorded. No visible changes were observed.

#### Imaging

Image acquisition time was 100 ms per pixel and laser power 10 to 18 mW. Image dimensions, frame integration and stage step size were adjusted depending on the image type: 30 by 200 pixels, two frames, and 5 μm for root tips; two frames and 1 μm for the different developmental regions in the cortex layer and root hairs (variable pixel dimensions); and 100 by 10 pixels, four frames, and 1 μm for the elongation zone (four- and six-day-old seedlings, mutants and treatments).

#### Spectral resolution

According to the manufacturer specifications (Cobolt), the laser linewidth is <1 MHz. The spectral resolution of the spectrometer, which defines the ability to distinguish light from different wavelengths (*60*), was determined by measuring the width of an elastically scattered peak, from the same orders (between second and third order) as the ones used to measure samples, reflected from a flat mirror (fig. S10B). The detected laser peak (blue circles) was fitted with a Lorentzian function (red curve). The linewidth (full width at half maximum, FWHM) of the fitted peak was 33.2 pixels, corresponding to 488 MHz, after converting pixels to Hz using water and methanol as calibration samples. We calculated the finesse as the ratio between the free spectral range and the FWHM and obtained a value of 64.

#### Spatial resolution

The spatial resolution of a Brillouin microscope depends on the attenuation length of the collective excitations (phonons), which is sample dependent (*65*). Scanning across the interface between polymethyl methacrylate and immersion oil type F (Leica, refractive index *n* = 1.518) was performed to quantify the spatial resolution. Data along the interface were fitted with an erf function and its derivative was obtained to calculate the FWHM. A lateral resolution of 2 μm and an axial resolution of 8 μm was obtained (fig. S10, C and D).

#### Spectral precision

To determine how accurately and reproducibly our frequency measurements are, we repeatedly measured the frequency shift and linewidth of distilled water (fig. S10, E and F) and calculated the SD from our measurements. The data are represented as a histogram and fitted with a Gaussian curve. We obtained a precision of 10 MHz for the frequency shift and 20 MHz for the linewidth using 10 mW laser power.

#### Image analysis

A custom MATLAB (MathWorks) script was used to extract the Brillouin peaks position and linewidth (FWHM) of the Brillouin peaks (fig. S11) after background subtraction and fitting the peaks with a Lorentzian function

$$L\left(A,x_{0},x,G\right)=\frac{A}{p}\frac{\frac{1}{2}G}{{\left(x-x_{0}\right)}^{2}+\frac{1}{2}G}$$ (1)

where *A* is the intensity, *x*_0_ is the center of the function, *x* is a variable representing the position and *G* is a parameter that specifies the width of the function. We performed deconvolution by subtracting the FWHM from the Raileigh peak (also assumed to have a Lorentzian shape) from the measured FWHM.

Peak positions obtained from distilled water and methanol served as reference samples and were used to calibrate the frequency axis by obtaining the pixel to frequency equivalency and FSR. Calibration was performed using the values of water (ν_B_ = 7.45 GHz) and methanol (ν_B_ = 5.45 GHz) with a 532-nm laser wavelength.

Regular measurements of water and methanol were performed and interpolation between measurement time points was used to assign the corresponding calibration values.

Calculated frequency shift and linewidth values were ordered according to image dimensions to generate the corresponding heatmaps. Image overlays between confocal images and heatmaps were done manually in Adobe Illustrator, which were subsequently used for defining the region of interest for quantification.

#### Calculation of the mass density from refractive index

The refractive index data of root hair peripheries obtained using QPI was used to estimate the mass density. The sample was considered as a two-substance mixture of dry and fluid fraction, and the mass density was calculated as (*44*)

ρ≈n−nfluiddndc+ρfluid (2)

where ρ is the mass density of the sample, *n* is the refractive index of the sample, *n*_fluid_ is the refractive index of the fluid fraction (refractive index of distilled water *n* = 1.33), *dn/dc* is the refraction increment [~0.145 ml/g for carbohydrates; (*66*)], and ρ_fluid_ is the mass density of the fluid fraction (1 g/ml for water).

#### Calculation of the longitudinal modulus

The longitudinal modulus is composed of two terms (*17*, *20*): the real part *M*′ (longitudinal storage modulus), which accounts for the elastic behavior, and the imaginary part *M*″ (longitudinal loss modulus), which accounts for the sample’s attenuation properties, reflecting on its viscous behavior.

The frequency shift serves as a proxy for the elastic behavior of a material as it relates to the longitudinal modulus of by the formula (with a scattering collection angle of 180°)

$$M^{\prime }=\rho {\left(\frac{\lambda }{2n}\right)}^{2}\nu_{B}^{2}$$ (3)

where ν_B_ is the frequency shift, *n* is the refractive index, λ is the excitation laser frequency, *M*′ is the real part of the longitudinal modulus, and ρ is the material density.

The linewidth is used as a proxy for the longitudinal viscosity of a material. They are related by the formula

$$M^{\prime \prime }=\rho {\left(\frac{\lambda }{2n}\right)}^{2}\nu_{B}\Delta_{B}$$ (4)

where ν_B_ is the frequency shift, *n* is the refractive index, λ is the excitation laser frequency, *M*″ is the imaginary part of the longitudinal modulus (loss modulus), and ρ is the material density. The longitudinal viscosity includes the effects of both shear and bulk viscosity (*17*, *60*).

### Data processing, visualization, and statistical analysis

The data were compiled with R and visualized using the ggplot2 package. Data normality was tested using the Shapiro-Wilk method (α = 0.05).

#### Root tip (Brillouin microscopy)

Data were collected as a list of values starting at the position above the stem cell niche and ending at the elongation zone for every sample (Fig. 1). Each value in the list representing the mean of 5 pixels at each position along the root tissue, ensuring that each pixel set contains values for cell walls and cytoplasmic regions in all root zones. Individual data points, representing the mean of all samples (mean of the lists values) at each position, were plotted for all tissues, starting from the position above the stem cell niche (Fig. 1C). Data points were fitted using a generalized additive model visualized as solid curves with SE. A solid vertical line indicates the position of the transition zone based on the first elongated cell in the cortex file. Between 9 and 12 roots were measured for each tissue.

#### Root tip (FLIM)

After analysis was completed in SymphoTime 64 (PicoQuant, Germany), lifetime data were saved and imported as a text image into ImageJ (fig. S1A). Cell walls of individual tissue layers were selected with a segmented line, and a list of the longitudinal lifetime values was obtained using the PlotProfile function. After averaging the values for each tissue, the data were fitted using loess smoothing, visualized as solid curves with SE. A solid vertical line indicates the position of the transition zone based on the first elongated cell in the cortex file. The cortex, endodermis and stele tissues of 10 roots were analyzed.

#### Longitudinal and transversal cell wall measurements in the cortex (Brillouin microscopy)

For each measured cell, data were collected by selecting a trajectory with the maximum pixel value along the two longitudinal or two transversal cell walls (Fig. 2 and fig. S2). Data points represent the mean value of each cell wall type (Fig. 2, C and E). Boxplots were used to visualize the data where measurements from a single cell (longitudinal and transversal cell walls) were paired. A paired *t* test was used to evaluate significant differences between the measurements of different cell wall orientations. Between 8 and 11 cells in Col-0 and between 7 and 9 in each mutant were measured for each developmental zone.

#### Longitudinal and transversal cell wall measurements in the cortex (FLIM)

After analysis of an image was completed in SymphoTime 64 (PicoQuant, Germany), entire longitudinal or transversal cell walls were selected using the free selection tool (fig. S1B). The exponential fit and lifetime values were recalculated for the region of interest. Boxplots were used to visualize the data where measurements from a single cell (longitudinal and transversal cell walls) were paired. A paired *t* test was used to evaluate significant differences between the measurements of different cell wall orientations. Ten cells were measured for each developmental zone.

#### Elongation zone

Data were collected by selecting a trajectory of pixels with the maximum values along cell walls and calculating the mean (10 pixels for each root; Fig. 3, D and E) (Fig. 3 and fig. S5). Pixels between adjacent cell walls trajectories were used to calculate the cytoplasmic values. Because cell wall and cytoplasm regions in the stele were indistinguishable, the whole stele region was selected to calculate the mean frequency shift and linewidth values. Box plots were used to represent paired data points of the different root tissues. A repeated-measures analysis of variance (ANOVA) test with Holm correction was used to estimate significant differences among tissues. Ten roots were measured for each tissue.

#### Mutants elongation zone

Data were collected and visualized in the same manner as for the elongation zone data (Fig. 4 and fig. S6). ANOVA with Dunnet’s post hoc test was performed to determine significant differences in mutant vs Col-0 (reference group) measurements for each tissue. Between 9 and 11 roots were analyzed for each mutant line. Nineteen roots were analyzed for Col-0.

#### Treatments

Data were collected and visualized in the same manner as for the elongation zone data (Fig. 5 and figs. S7 to S9). ANOVA with Dunnet’s post hoc test was performed to determine significant differences in treated vs control (reference group) measurements for each tissue. Between 15 and 23 roots were analyzed for Col-0 and between 9 and 12 for the mutants.

#### Root hairs

Width and length of root hairs are visualized in fig. S3B. Each dot represents an individual root hair from a different root. To quantify the mechanical properties, root hair cells were divided into base, middle, and tip regions. High-value pixels of ellipsoidal regions at the base, likely belonging to nuclei, were excluded from the analysis. Each root hair was represented as a set of three paired data points, one in each region (fig. S2E). A repeated-measures ANOVA test with pair-wise *t* test and Bonferroni correction was used to determine significant differences among the root hair regions. Quantification was done in 19 root hairs from different roots.

#### Nuclei and cytoplasm in hypocotyls, roots and root hairs (Brillouin microscopy)

Data represents the average of the nucleus and cytoplasmic pixels for each cell measured (fig. S4). A paired *t* test was used to determine significant differences. Samples sizes were *n* = 6 for roots and root hairs and *n* = 8 for hypocotyls. *P* values are reported as ns > 0.05, * < 0.05, ** < 0.01, *** < 0.001, **** < 0.0001.

## Supplementary Materials

### The PDF file includes:

Figs. S1 to S11 Table S1 Legends for data scripts S1 to S3 References

### Other Supplementary Material for this manuscript includes the following:

Data Scripts S1 to S3
