# Supplementary material for: The mechanical properties of Arabidopsis thaliana roots adapt dynamically during development and to stress
Source: Sci Adv. 2026 Feb 18;12(8):eaeb0032. doi: 10.1126/sciadv.aeb0032 (PMC12915607; doi:10.1126/sciadv.aeb0032)
Supplement: Supplementary file 1 — Figs. S1 to S11 Table S1 Legends for data scripts S1 to S3 References [file sciadv.aeb0032_sm.pdf]

Science Advances
AAAS

Supplementary Materials for

The mechanical properties of *Arabidopsis thaliana* roots adapt dynamically during development and to stress

Luis Alonso Baez *et al.*

Corresponding author: Thorsten Hamann, thorsten.hamann@ntnu.no

Sci. Adv. **12**, eaeb0032 (2026)
DOI: 10.1126/sciadv.aeb0032

The PDF file includes:

Figs. S1 to S11
Table S1
Legends for data scripts S1 to S3
References

Other Supplementary Material for this manuscript includes the following:

Data Scripts S1 to S3

Fig. S1.

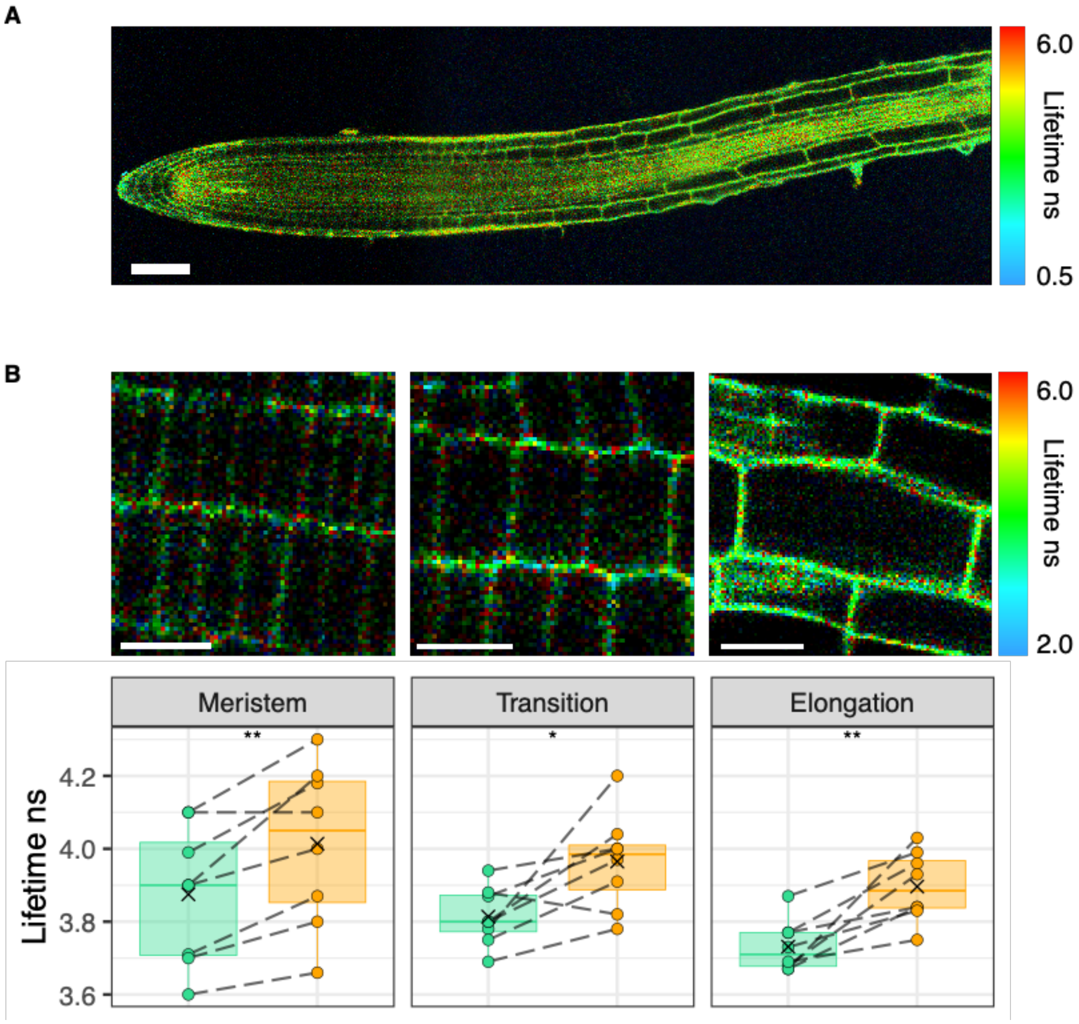

Fig. S1 Measurement of cell wall porosity using CarboTag-BDP molecular rotors. Fluorescence lifetime images of (A) the root tip (n = 10, scale bar 100 μm) and (B) (Top) distinct developmental zones along the cortex layer. Scale bars represent 20 μm for the meristem and transition zone and 30 μm for the elongation zone. (Bottom) Quantification of the fluorescence lifetime in longitudinal (green data) and transversal cell walls (orange data) in every zone. n = 10 cells from different roots for each root zone. A paired t-test was used to obtain the statistics for each root zone in (B).

Fig. S2.

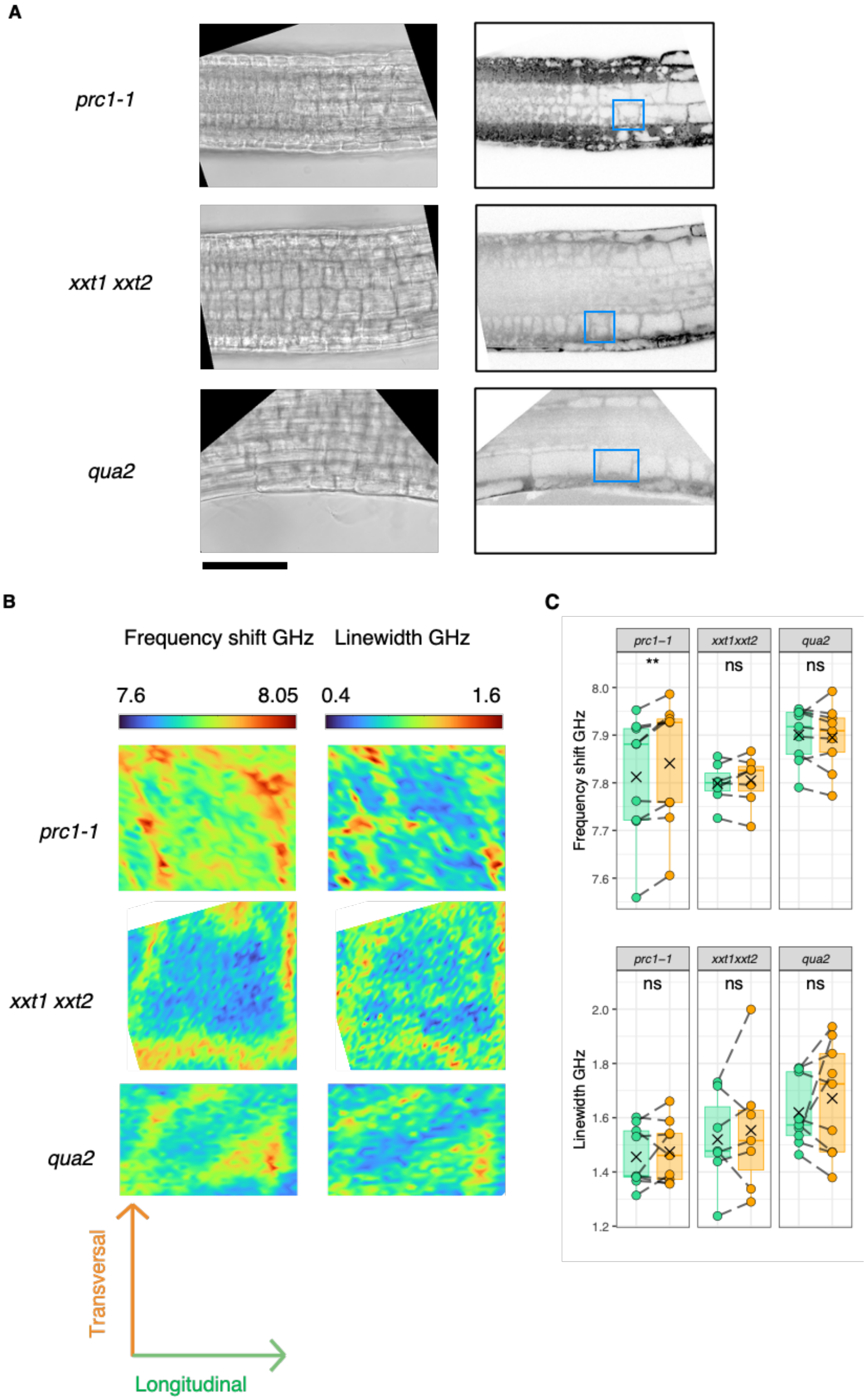

Fig S2. Cell wall anisotropy of cortex cells located in the transition zone of selected mutants. (A) Brightfield (left) and fluorescence (right) images of the corresponding mutants. Scale bar 100 μm. (B) Maps of the frequency shift (left) and linewidth (right) of *prc1-1* (35 x 35

μm), *xxt1xxt2* (35 x 35 μm) and *qua2* (35 x 50 μm). (C) Quantification of the frequency shift (top) and linewidth (bottom) of longitudinal (green) and transversal (orange) cell walls, n = 7-9 cells from different roots. A paired t-test was used to obtain the statistics.
Type or paste caption here. Create a page break and paste in the Figure above the caption.

Fig. S3.

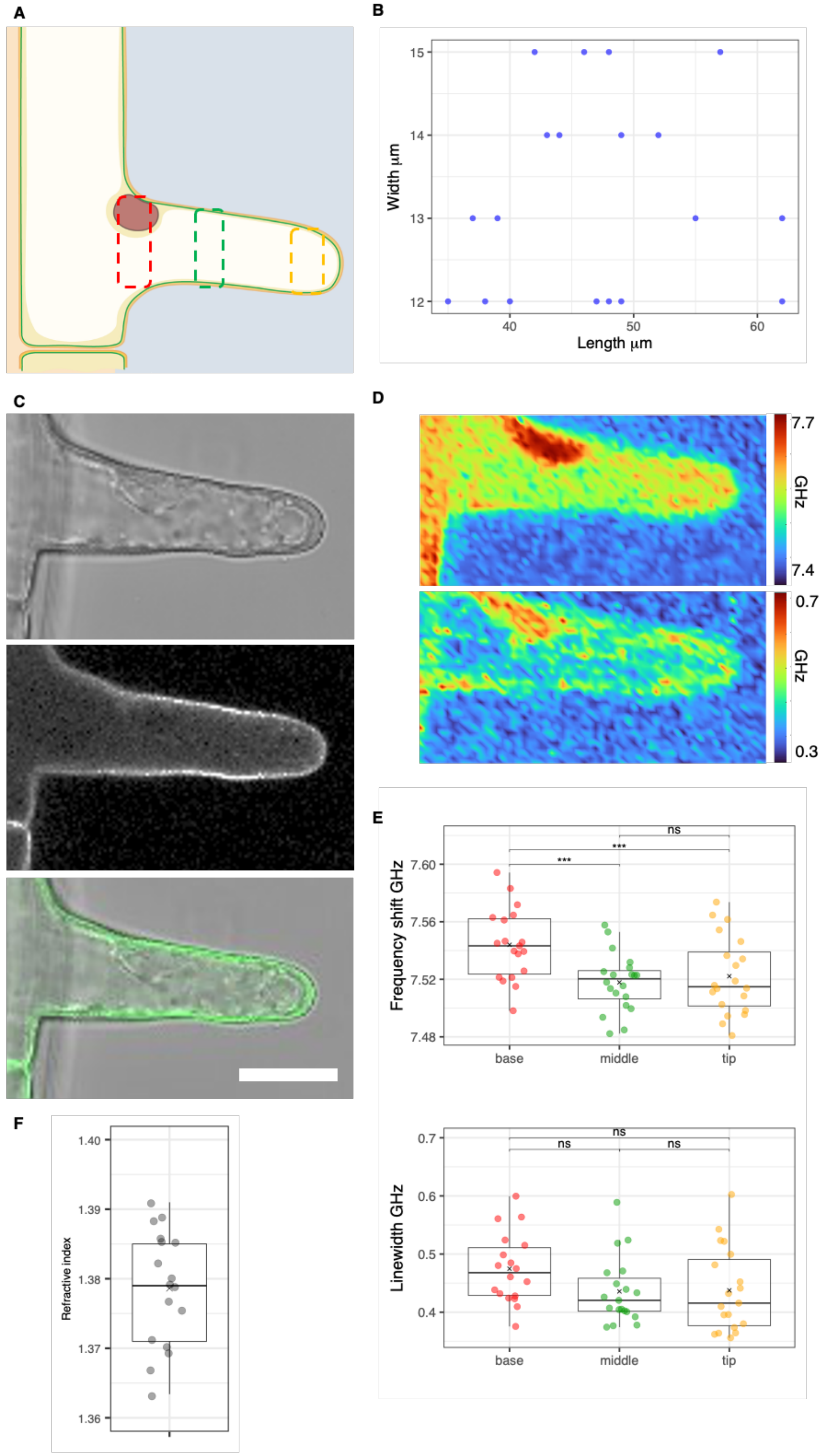

A
B
Width µm
Length µm
15
14
13
12
40
50
60
C
D
7.7
GHz
7.4
0.7
GHz
0.3
E
Frequency shift GHz
7.60
7.56
7.52
7.48
ns
***
***
base
middle
tip
Linewidth GHz
0.7
0.6
0.5
0.4
ns
ns
ns
base
middle
tip
F
Refractive index
1.40
1.39
1.38
1.37
1.36

Fig. S3 Mechanical properties of root hairs. (A) Schematic representation of a developing root hair. Regions used for quantification are marked by dashed rectangles. (B) Length and width of analysed root hairs Every blue dot represents a root hair from a different root. (C) Brightfield (top), confocal image with p35S::LTI6b-GFP plasma membrane marker (middle), overlap (bottom). Scale bar 20 µm. (D) Heatmaps (35 x 70 µm) of the frequency shift (top) and linewidth (bottom). (E) Quantification of frequency shift (top) and linewidth (bottom) at base, middle and tip regions of root hairs. (F) Quantification of the refractive index at root hair peripheries. n = 19 root hairs. A repeated measures ANOVA with pair-wise t-test and Bonferroni correction was used to obtain the statistics.

Fig. S4

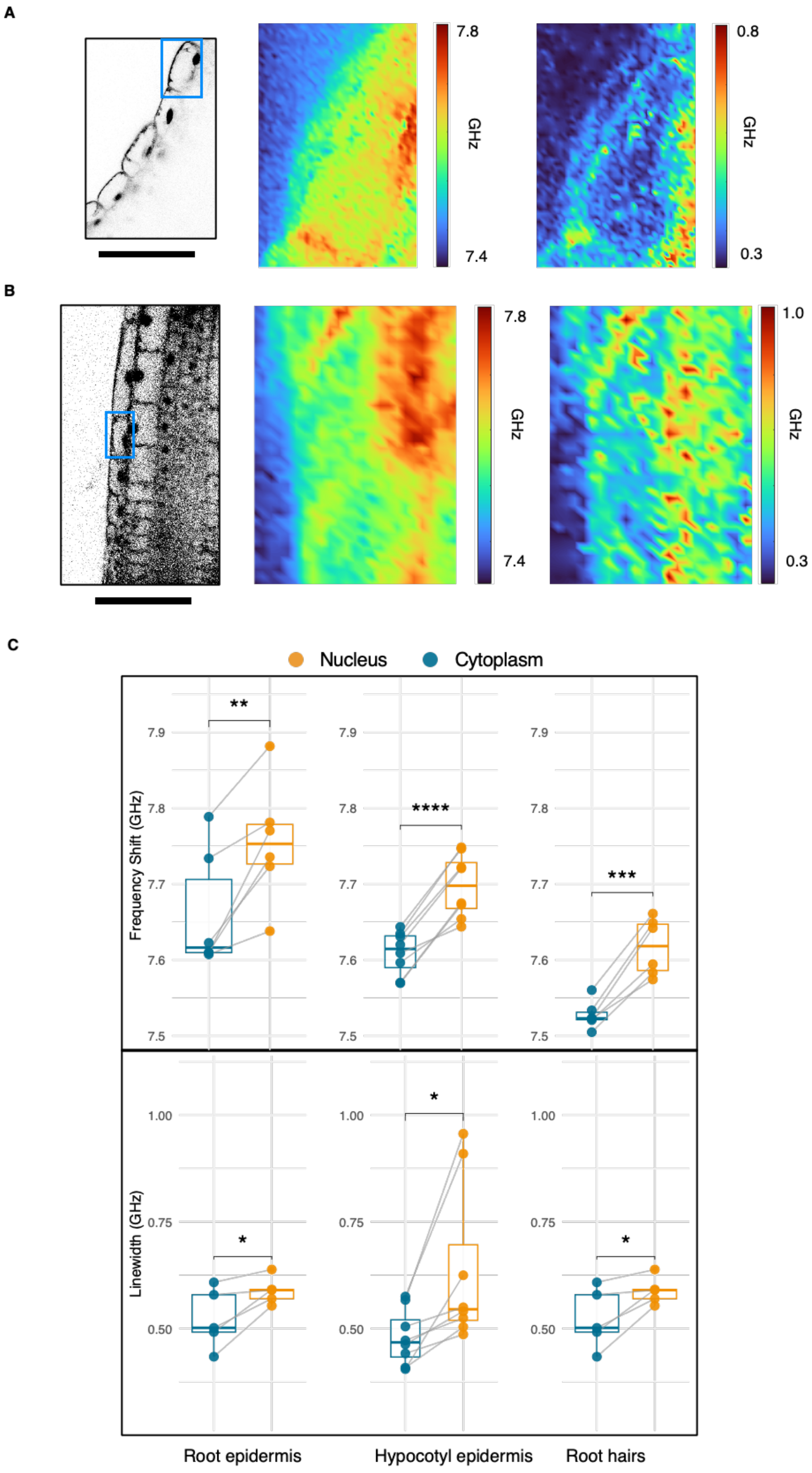
A
7.8
7.4
GHz
0.8
0.3
GHz
B
7.8
7.4
GHz
1.0
0.3
GHz
C
Nucleus
Cytoplasm
Frequency Shift (GHz)
7.9
7.8
7.7
7.6
7.5
**
****
***
Linewidth (GHz)
1.00
0.75
0.50
*
*
*
Root epidermis
Hypocotyl epidermis
Root hairs

Fig. S4 Mechanical properties of the nucleus. The p35S::NLS-GFP line was used to identify the position of the nucleus. (A) Hypocotyl and (B) root images based on fluorescence (left), frequency shift (middle) and linewidth (right). Brillouin image sizes are 60x40 µm for the hypocotyl and 40x25 µm for the root epidermis. Scale bar 100 µm. (C) Quantification of frequency shift and linewidth in the nucleus and cytoplasm of root epidermis, hypocotyls and root hairs. $n_{root} = 6$, $n_{hypocotyl} = 8$ and $n_{roothair} = 6$. A paired t-test was used to perform statistical testing.

Fig. S5

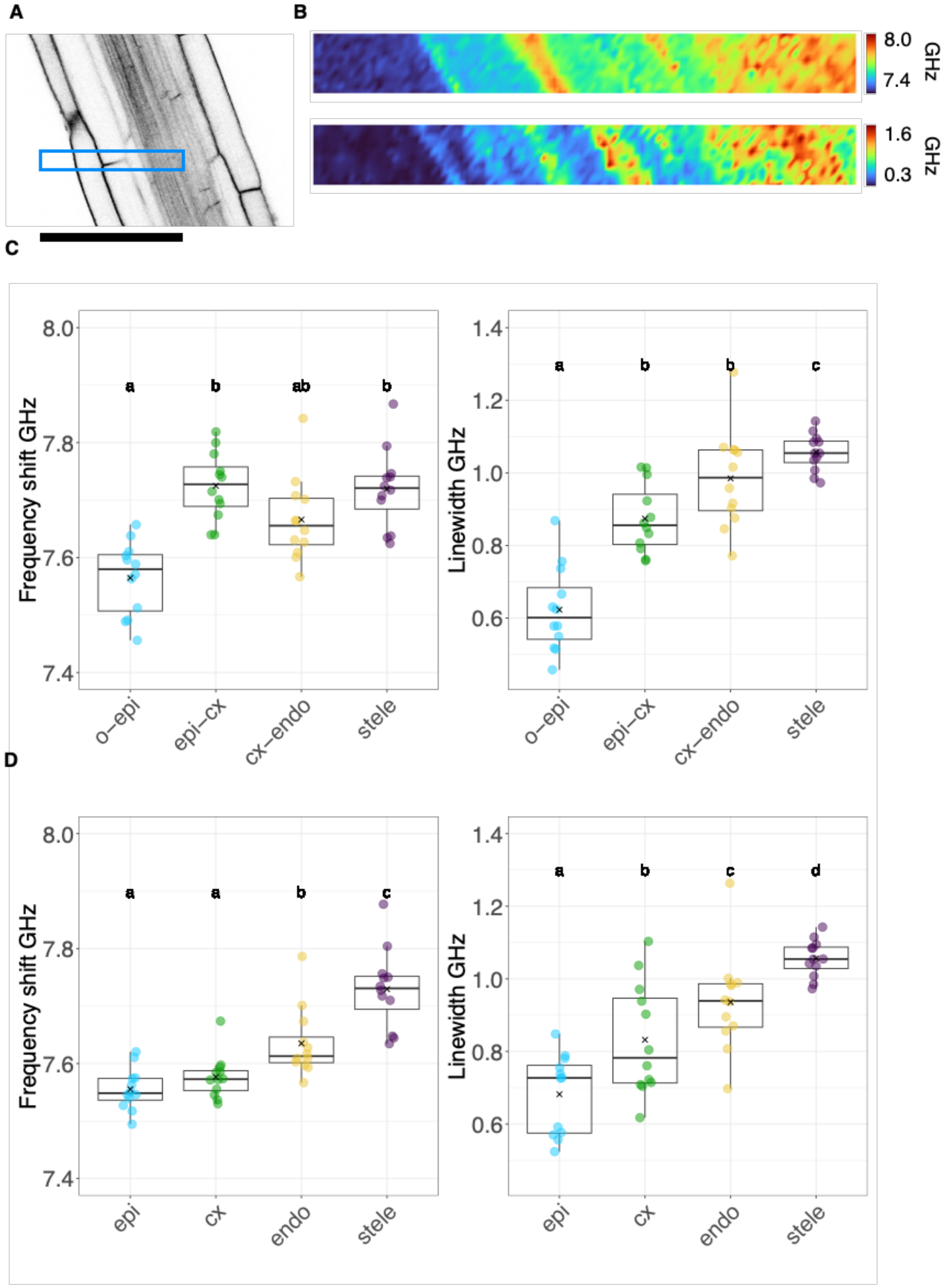

Fig. S5. Mechanical properties in the elongation zone of 4-days-old seedlings. (A) Confocal image of the root elongation zone with p35S::LTI6b-GFP. Scale bar 100 µm. (B) Heatmaps of the mechanical properties in the elongation zone tissues (10 x 100 µm), frequency shift (top) and

linewidth (bottom). (C) Quantification of the mechanical properties in cell walls. (D) Quantification of the mechanical properties in cytoplasm. (C-D) Frequency shift (left) and linewidth (right). n = 11 roots. A repeated measures ANOVA with Holm correction was used to obtain the statistics. O = outside, epi = epidermis, cx = cortex, endo = endodermis.

Fig. S6

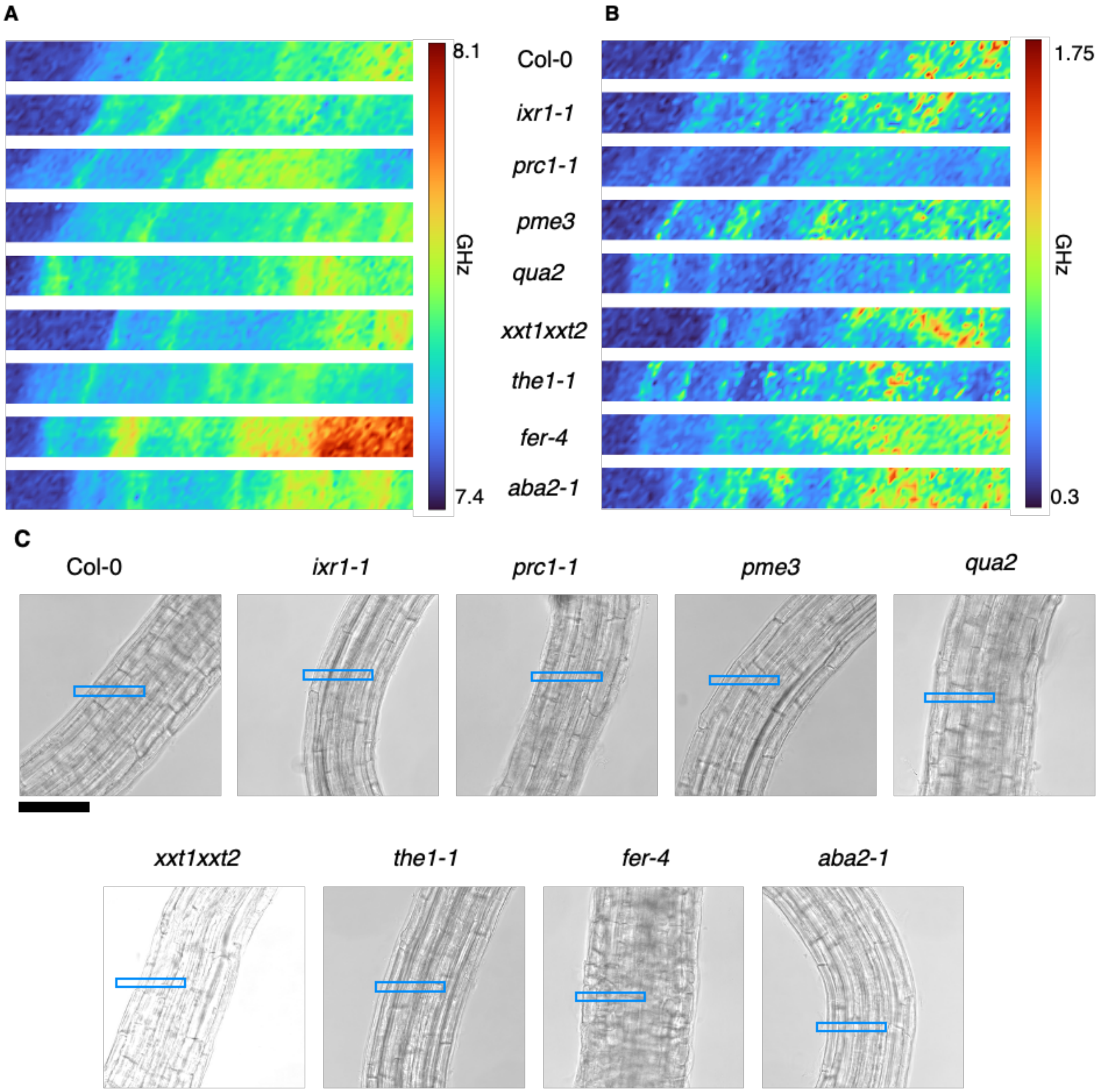

Fig. S6. Mechanical properties in the root elongation zone in mutants affected in cell wall metabolism, cell wall integrity signaling or ABA biosynthesis. Heatmaps of (A) frequency shift and (B) linewidth in the selected mutants (10x100 µm). (C) Brightfield images of the elongation zone of the respective mutants. Scale bar 100 μm.

Fig. S7

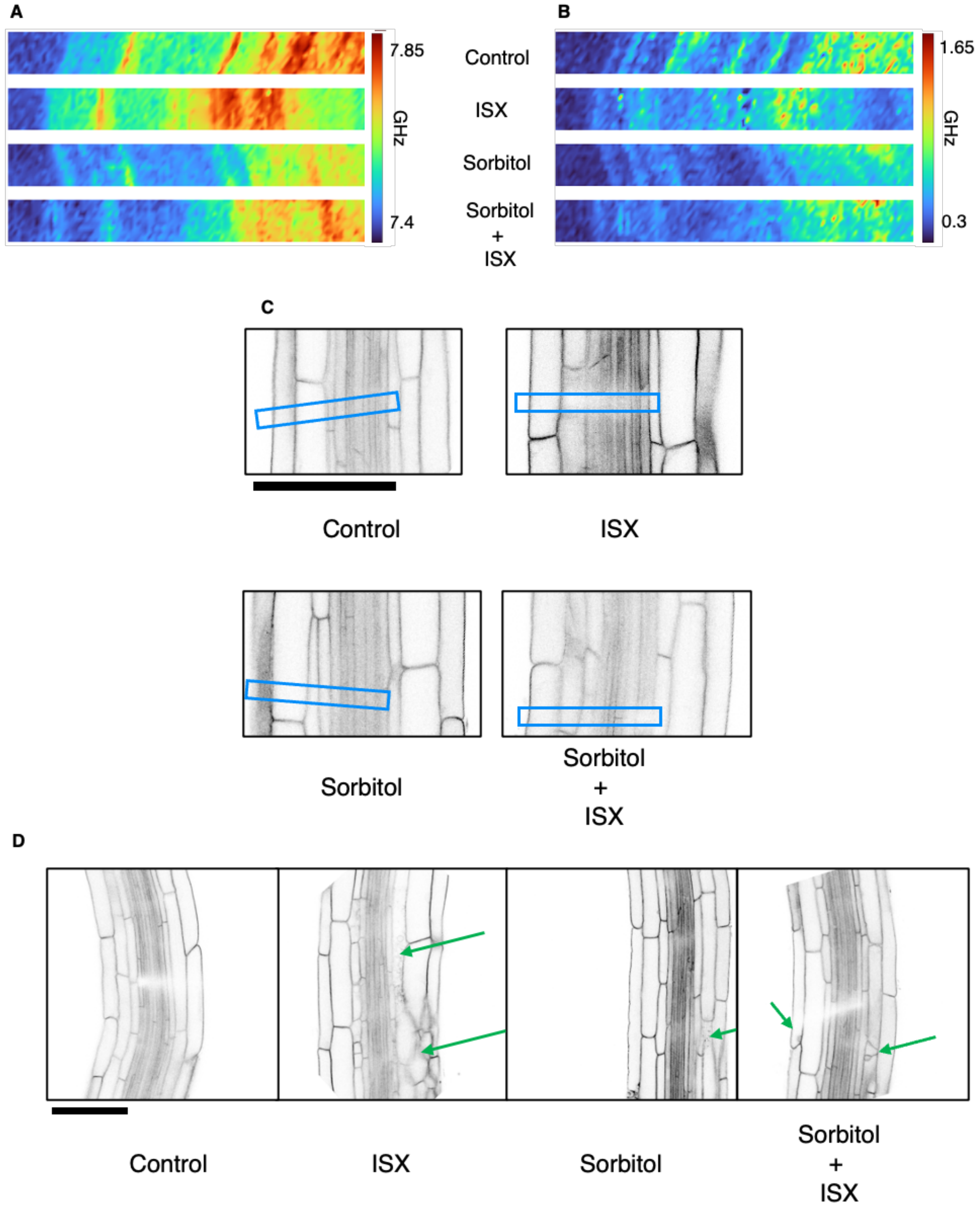

Fig. S7. Mechanical properties of tissue layers in the root elongation zone in response to different stresses. Heatmaps (10 x 100 μm) of the (A) frequency shift and (B) linewidth. (C) Confocal images with plasma membrane marker p35S::LTI6b-GFP. (D) Effects of 3h stress conditions highlighted with green arrows. Scale bars 100 μm

Fig. S8

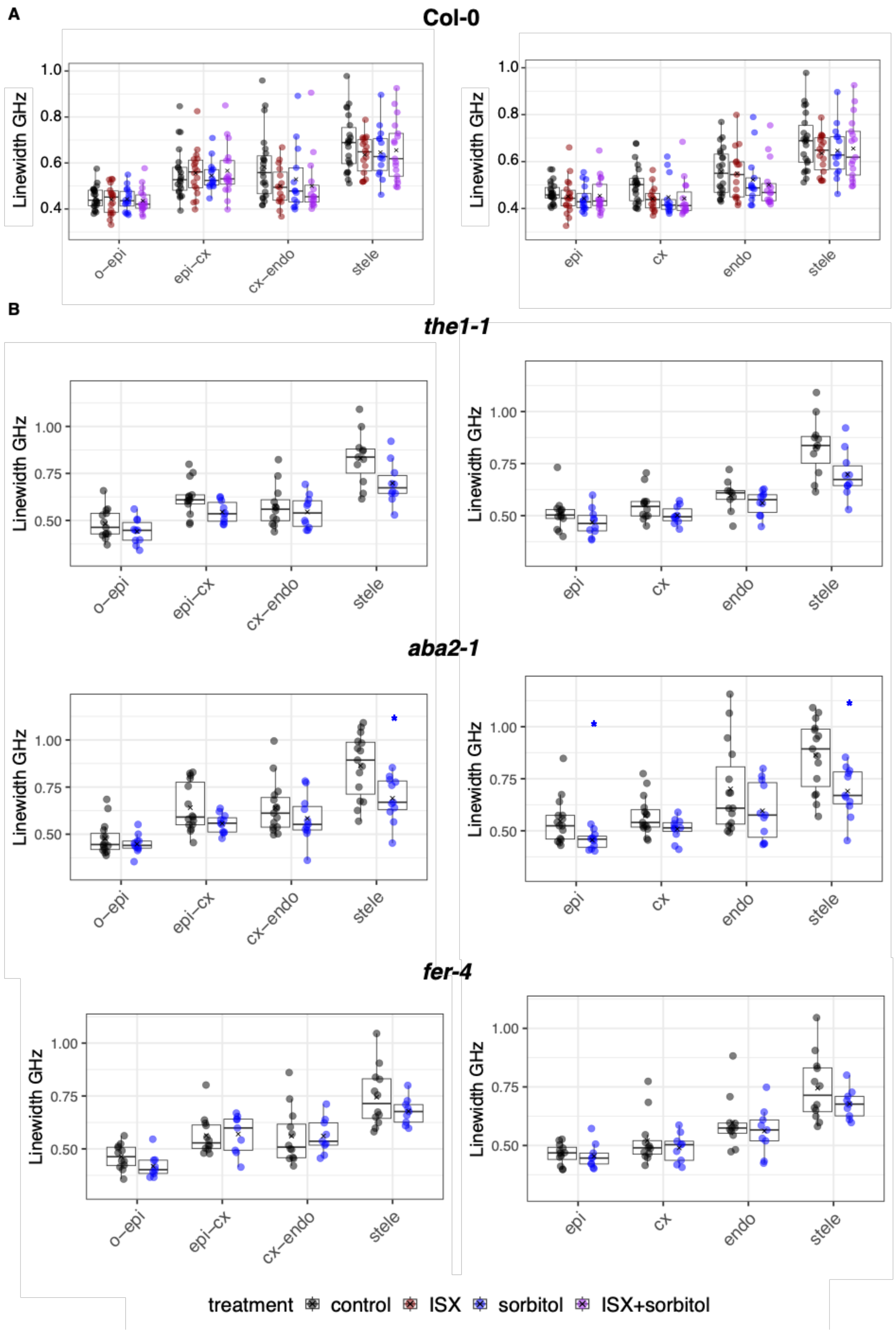

Fig S8. Linewidth in the elongation zone in response to stress. Quantification of the linewith in cell walls (left column) and cytoplasm (right column) in (A) Col-0 and (B) selected mutants.

$n_{Col\text{-}0}$ = 15-23 roots and $n_{mutants}$ = 9-12 roots. A one-way ANOVA with Dunnet's post hoc test was used to obtain the statistics. ISX = isoxaben. O = outside, epi = epidermis, cx = cortex, endo = endodermis.

Fig. S9

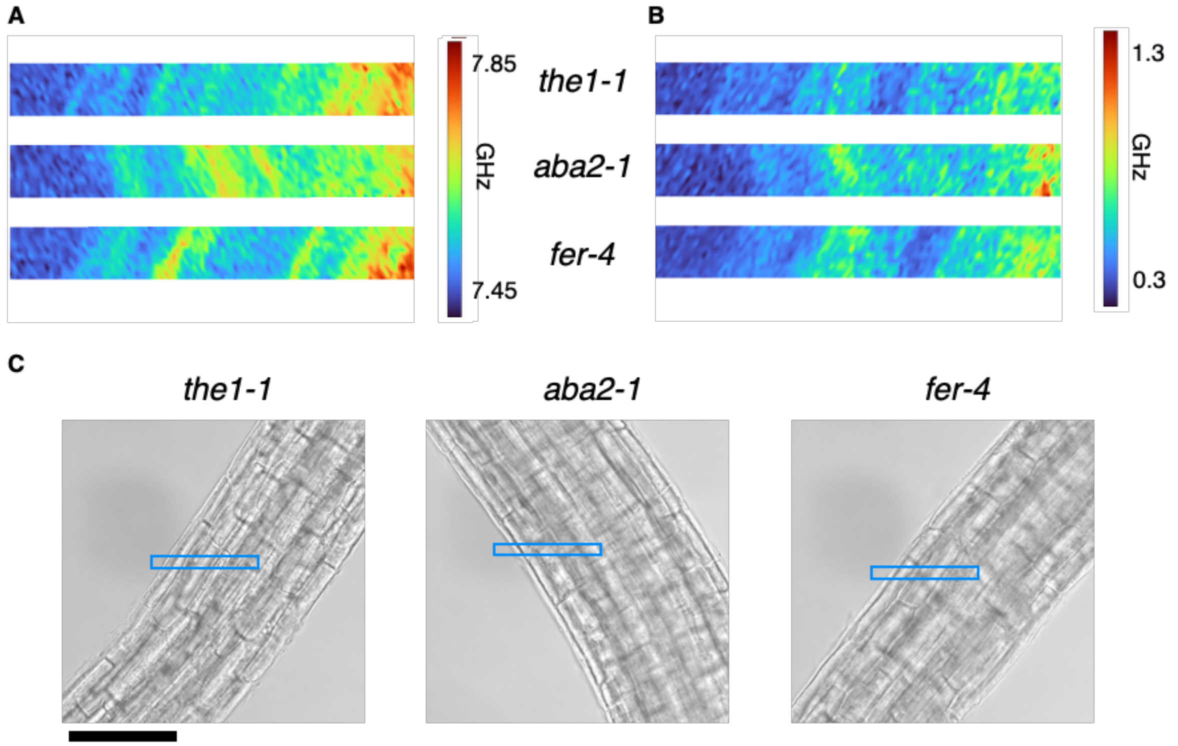

Fig. S9 Mechanical properties of selected mutants exposed to sorbitol. Heatmaps (10 x 100 μm) of the (A) frequency shift and (B) linewidth. (C) Brightfield images of the elongation zone. Scale bar 100 μm.

Fig. S10

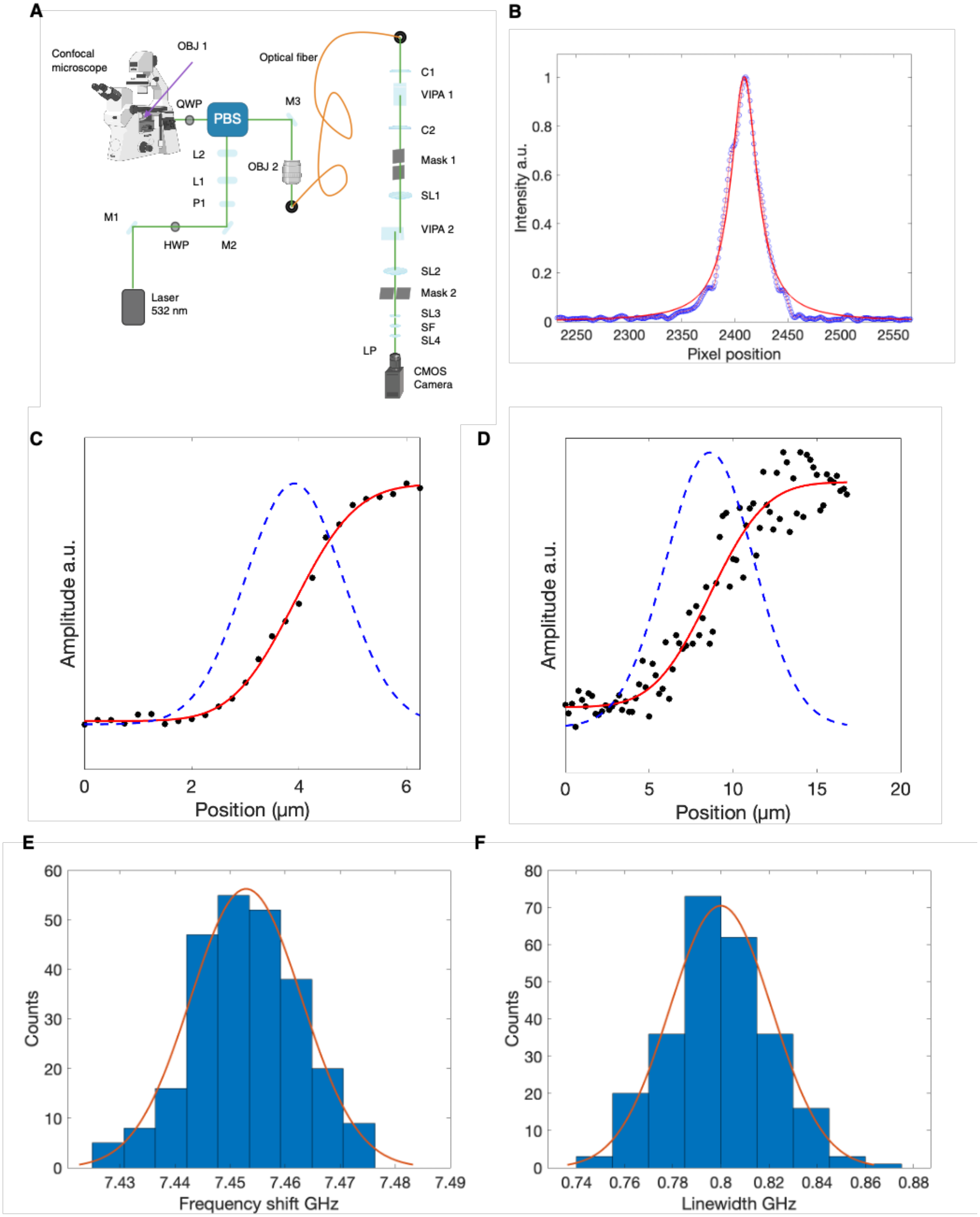

A
Confocal microscope
OBJ 1
Optical fiber
QWP
PBS
M3
L2
OBJ 2
L1
P1
M1
HWP
M2
Laser 532 nm
C1
VIPA 1
C2
Mask 1
SL1
VIPA 2
SL2
Mask 2
SL3
SF
SL4
LP
CMOS Camera
B
Intensity a.u.
Pixel position
C
Amplitude a.u.
Position (µm)
D
Amplitude a.u.
Position (µm)
E
Counts
Frequency shift GHz
F
Counts
Linewidth GHz

Fig. S10. Brillouin microscope instrumentation and resolution. (A) Schematic of the Brillouin microscope setup. M1 = mirror 1, HWP = half-wave plate, M2 = mirror 2, P1 = polarizer 1, L1 = beam expander lens 1, L2 = beam expander lens 2, PBS = polarized beam splitter, QWP = quarter-wave plate, OBJ1 = objective 1, M3 = mirror 3, OBJ2 = objective 2, C1 = cylindrical lens 1, VIPA1 = virtually imaged phased array 1, C2 = cylindrical lens 2, SL1 = spherical lens 1, VIPA2 = virtually imaged phased array 2, SL2 = spherical lens 2, SL3 = spherical lens 3, SF= spatial filter, SP4 = spherical lense 4, LP = lens pair. Created in BioRender. Ticha, M. (2025) https://BioRender.com/9xvw9rk. (B) Spectral resolution determination by fitting a Lorentzian function (red curve) to Rayleigh peak data (blue open circles).(C) Lateral and (D) axial resolution measurement across an interface between oil and polumethyl methacrylate. Black dots represent data points, *erf* fit in red and its derivative in dashed blue line. Histogram of repeated (E) frequency shift and (F) linewidth measurements on distilled water to calculate the instrument spectral precision. A Gaussian fit (red curve) was used to calculate the FWHM from the data (blue bars).

Fig. S11

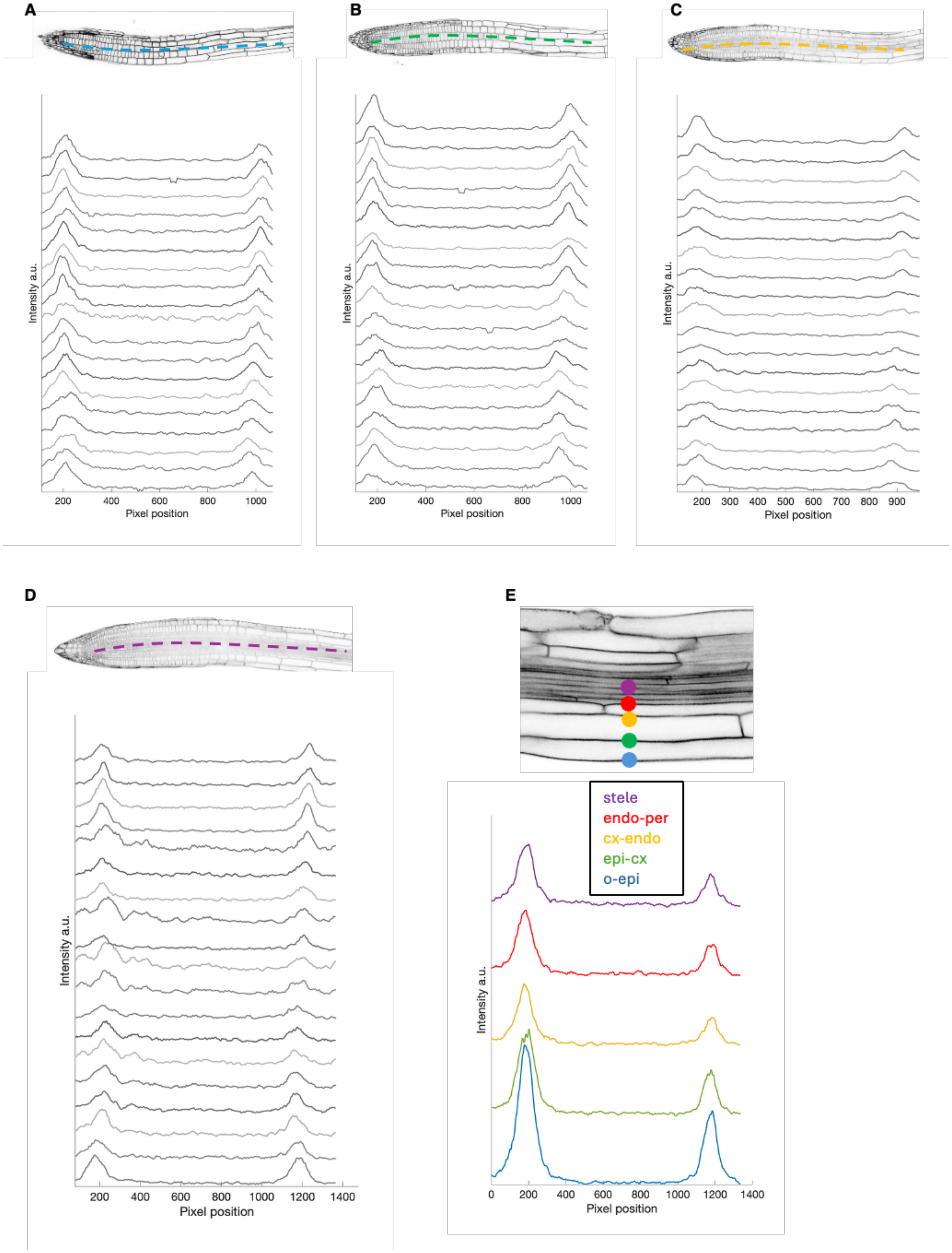
A
B
C
D
E
Intensity a.u.
Pixel position
200
400
600
800
1000
300
500
700
900
1200
1400
0
stele
endo-per
cx-endo
epi-cx
o-epi

Fig. S11. Brillouin spectra from different tissues. The Brillouin spectra were visually inspected to verify the quality of the Brillouin peaks and check for noise levels. For each tissue plane of Fig. 1, spectra located every 20 μm along developmental axes (colored dashed lines in the root) were plotted as representative examples of (A) epidermis, (B) cortex, (C) endodermis and (D) stele tissues. (E) Spectra localized at cell wall positions of the elongation zone (colored dots), corresponding to Fig. 3.

Table S1.

AGI code	Genotype	Background	Source
-	Col-0		NASC
-	p35S-LTI6b-GFP	Col-0	Benoit Landrein (École Normale Supérieure Lyon)
At5g05170	*ixr1-1*	Col-0	Chris Sommerville (UC Berkeley)
At5g64740	*prc1-1*	Col-0	Cyril Zipfel (University of Zürich)
At3g14310	*pme3*<br>N400106	Col-0	Timo Engelsdolf (University of Marburg)
At3g62720<br>At4g02500	*xxt1 xxt2*	Col-0	NASC
At5g54380	*the1-1*	Col-0	Herman Höfte (INRAE Versailles)
At3g51550	*fer-4*	Col-0	Malcolm Bennett (University of Nottingham)
At2g03480	*qua2*	Col-0	Charles Anderson (Pennsylvania University)
At1g52340	*aba2-1*	Col-0	(*67*)

Table S1. Arabidopsis lines used in this study.

Data Scripts.

MATLAB scripts used for the analysis of Brillouin microscopy experiments.

Data Script file S1. Calculation of parameters in reference samples.

Data Script file S2. Calculation of the frequency shift and linewidth of test samples.

Data Script file S3. Saves the values obtained in **Data Script file S2** as a txt file.

1. **Data Script file S1**is run first to obtain the free spectral range (fsr) and pixel-to-frequency ratio (pr) from the reference samples.

2. Then, the **Data Script file S2** runs with the folder containing the Brillouin spectra images of the experiment.

3. Finally, **Data Script file S3** saves the values of the generated heatmaps as tables.

REFERENCES

1. R. Kelly-Bellow, K. Lee, R. Kennaway, J. E. Barclay, A. Whibley, C. Bushell, J. Spooner, M. Yu, P. Brett, B. Kular, S. Cheng, J. Chu, T. Xu, B. Lane, J. Fitzsimons, Y. Xue, R. S. Smith, C. D. Whitewoods, E. Coen, Brassinosteroid coordinates cell layer interactions in plants via cell wall and tissue mechanics. *Science* **380**, 1275–1281 (2023).

2. A. Bonfanti, E. T. Smithers, M. Bourdon, A. Guyon, P. Carella, R. Carter, R. Wightman, S. Schornack, H. Jönsson, S. Robinson, Stiffness transitions in new walls post-cell division differ between *Marchantia polymorpha* gemmae and *Arabidopsis* thaliana leaves. *Proc. Natl. Acad. Sci. U.S.A.* **120**, e2302985120 (2023).

3. T.-H. Yang, A. Che´telat, A. Kurenda, E. E. Farmer, Mechanosensation in leaf veins. *Sci. Adv.* **9**, eadh5078 (2023).

4. S. R. Silveira, L. Collet, S. M. Haque, L. Lapierre, A. Bagniewska-Zadworna, R. S. Smith, F. P. Gosselin, A.-L. Routier-Kierzkowska, D. Kierzkowski, Mechanical interactions between tissue layers underlie plant morphogenesis. *Nat. Plants* **11**, 909–923 (2025).

5. D.-C. Trinh, J. Alonso-Serra, M. Asaoka, L. Colin, M. Cortes, A. Malivert, S. Takatani, F. Zhao, J. Traas, C. Trehin, O. Hamant, How mechanical forces shape plant organs. *Curr. Biol.* **31**, R143–R159 (2021).

6. L. Hoermayer, J. C. Montesinos, N. Trozzi, L. Spona, S. Yoshida, P. Marhava, S. Caballero-Mancebo, E. Benková, C.-P. Heisenberg, Y. Dagdas, M. Majda, J. Friml, Mechanical forces in plant tissue matrix orient cell divisions via microtubule stabilization. *Dev. Cell* **59**, 1333–1344. e4 (2024).

7. R. Goswami, A. Asnacios, O. Hamant, M.-E. Chabouté, Is the plant nucleus a mechanical rheostat? *Curr. Opin. Plant Biol.* **57**, 155–163 (2020).

8. L. Hong, M. Dumond, M. Zhu, S. Tsugawa, C.-B. Li, A. Boudaoud, O. Hamant, A. H. K. Roeder, Heterogeneity and robustness in plant morphogenesis: From cells to organs. *Annu. Rev. Plant Biol.* **69**, 469–495 (2018).

9. A. Walia, R. Carter, R. Wightman, E. M. Meyerowitz, H. Jönsson, A. M. Jones, Differential growth is an emergent property of mechanochemical feedback mechanisms in curved plant organs. *Dev. Cell* **59**, 3245–3258.e3 (2024).

10. B. P. Lapointe, N. S. Kaur, A.-L. Routier-Kierzkowska, A. Burian, From stress to growth: Mechanical tissue interactions in developing organs. *Curr. Opin. Plant Biol.* **86**, 102759 (2025).

11. S. Liu, S. Strauss, M. Adibi, G. Mosca, S. Yoshida, R. Dello Ioio, A. Runions, T. G. Andersen, G. Grossmann, P. Huijser, R. S. Smith, M. Tsiantis, Cytokinin promotes growth cessation in the Arabidopsis root. *Curr. Biol.* **32**, 1974–1985.e3 (2022).

12. A. Peaucelle, R. Wightman, H. Höfte, The control of growth symmetry breaking in the Arabidopsis hypocotyl. *Curr. Biol.* **25**, 1746–1752 (2015).

13. A. Sapala, R. S. Smith, "Osmotic Treatment for Quantifying Cell Wall Elasticity in the Sepal of *Arabidopsis thaliana*" in *Plant Stem Cells*, M. Naseem, T. Dandekar, Eds. (Springer US, New York, NY, 2020). vol. 2094, pp. 101–112.

14. F. Bou Daher, L. Serra, R. Carter, H. Jönsson, S. Robinson, E. M. Meyerowitz, W. M. Gray, Xyloglucan deficiency leads to a reduction in turgor pressure and changes in cell wall properties, affecting early seedling establishment. *Curr. Biol.* **34**, 2094–2106.e6 (2024).

15. N. Trozzi, W. Wodniok, R. Kelly-Bellow, A. Meraviglia, A. Chételat, N. Adkins, B. Lane, R. S. Smith, D. Kwiatkowska, M. Majda, Camelot: A computer-automated micro-extensometer with low-cost optical tracking. *BMC Biol.* **23**, 112 (2025).

16. L. Michels, V. Gorelova, Y. Harnvanichvech, J. W. Borst, B. Albada, D. Weijers, J. Sprakel, Complete microviscosity maps of living plant cells and tissues with a toolbox of targeting mechanoprobes. *Proc. Natl. Acad. Sci. U.S.A.* **117**, 18110–18118 (2020).

17. R. Prevedel, A. Diz-Muñoz, G. Ruocco, G. Antonacci, Brillouin microscopy: An emerging tool for mechanobiology. *Nat. Methods* **16**, 969–977 (2019).

18. H. Keshmiri, D. Cikes, M. Samalova, L. Schindler, L.-M. Appel, M. Urbanek, I. Yudushkin, D. Slade, W. J. Weninger, A. Peaucelle, J. Penninger, K. Elsayad, Brillouin light scattering anisotropy microscopy for imaging the viscoelastic anisotropy in living cells. *Nat. Photonics* **18**, 276–285 (2024).

19. M. Besten, M. Hendriksz, L. Michels, B. Charrier, E. Smakowska-Luzan, D. Weijers, J. W. Borst, J. Sprakel, CarboTag: A modular approach for live and functional imaging of plant cell walls. *Nat. Methods* **22**, 1081–1090 (2025).

20. I. Kabakova, J. Zhang, Y. Xiang, S. Caponi, A. Bilenca, J. Guck, G. Scarcelli, Brillouin microscopy. *Nat. Rev. Methods Primers* **4**, 8 (2024).

21. G. Antonacci, T. Beck, A. Bilenca, J. Czarske, K. Elsayad, J. Guck, K. Kim, B. Krug, F. Palombo, R. Prevedel, G. Scarcelli, Recent progress and current opinions in Brillouin microscopy for life science applications. *Biophys. Rev.* **12**, 615–624 (2020).

22. F. Palombo, D. Fioretto, Brillouin light scattering: Applications in biomedical sciences. *Chem. Rev.* **119**, 7833–7847 (2019).

23. M. Bailey, M. Alunni-Cardinali, N. Correa, S. Caponi, T. Holsgrove, H. Barr, N. Stone, C. P. Winlove, D. Fioretto, F. Palombo, Viscoelastic properties of biopolymer hydrogels determined by Brillouin spectroscopy: A probe of tissue micromechanics. *Sci. Adv.* **6**, eabc1937 (2020).

24. E. Coen, D. J. Cosgrove, The mechanics of plant morphogenesis. *Science* **379**, eade8055 (2023).

25. Y. Long, I. Cheddadi, G. Mosca, V. Mirabet, M. Dumond, A. Kiss, J. Traas, C. Godin, A. Boudaoud, Cellular heterogeneity in pressure and growth emerges from tissue topology and geometry. *Curr. Biol.* **30**, 1504–1516.e8 (2020).

26. Y. Zhang, J. Yu, X. Wang, D. M. Durachko, S. Zhang, D. J. Cosgrove, Molecular insights into the complex mechanics of plant epidermal cell walls. *Science* **372**, 706–711 (2021).

27. H. Oliveri, I. Cheddadi, Hydromechanical field theory of plant morphogenesis. *J. Mech. Phys. Solids* **196**, 106035 (2025).

28. O. E. Jensen, "Theoretical tools and concepts for modelling growing plant tissues" in *Soft Matter in Plants: From Biophysics to Biomimetics*, K. Jensen, Y. Forterre, Eds. (The Royal Society of Chemistry, 2022).

29. L. M. Di Fino, M. S. Anjam, M. Besten, A. Mentzelopoulou, V. Papadakis, N. Zahid, L. A. Baez, N. Trozzi, M. Majda, X. Ma, T. Hamann, J. Sprakel, P. N. Moschou, R. S. Smith, P. Marhavý, Cellular damage triggers mechano-chemical control of cell wall dynamics and patterned cell divisions in plant healing. *Dev. Cell* **60**, 1411–1422.e6 (2025).

30. Y. Fridman, S. Strauss, G. Horev, M. Ackerman-Lavert, A. Reiner-Benaim, B. Lane, R. S. Smith, S. Savaldi-Goldstein, The root meristem is shaped by brassinosteroid control of cell geometry. *Nat. Plants* **7**, 1475–1484 (2021).

31. R. Dello Ioio, F. S. Linhares, E. Scacchi, E. Casamitjana-Martinez, R. Heidstra, P. Costantino, S. Sabatini, Cytokinins determine Arabidopsis root-meristem size by controlling cell differentiation. *Curr. Biol.* **17**, 678–682 (2007).

32. D. De Vos, K. Vissenberg, J. Broeckhove, G. T. S. Beemster, Putting theory to the test: Which regulatory mechanisms can drive realistic growth of a root? *PLOS Comput. Biol.* **10**, e1003910 (2014).

33. R. Rahni, K. D. Birnbaum, Week-long imaging of cell divisions in the Arabidopsis root meristem. *Plant Methods* **15**, 30 (2019).

34. G. T. S. Beemster, T. I. Baskin, Analysis of cell division and elongation underlying the developmental acceleration of root growth in *Arabidopsis thaliana*. *Plant Physiol.* **116**, 1515–1526 (1998).

35. M. Majda, N. Trozzi, G. Mosca, R. S. Smith, How cell geometry and cellular patterning influence tissue stiffness. *Int. J. Mol. Sci.* **23**, 5651 (2022).

36. S. Persson, A. Paredez, A. Carroll, H. Palsdottir, M. Doblin, P. Poindexter, N. Khitrov, M. Auer, C. R. Somerville, Genetic evidence for three unique components in primary cell-wall cellulose synthase complexes in *Arabidopsis*. *Proc. Natl. Acad. Sci. U.S.A.* **104**, 15566–15571 (2007).

37. E. E. Sowinski, B. M. Westman, C. R. Redmond, Y. Kong, A. T. Olek, J. Olek, M. C. McCann, N. C. Carpita, Lack of xyloglucan in the cell walls of the Arabidopsis *xxt1/xxt2* mutant results in specific increases in homogalacturonan and glucomannan. *Plant J.* **110**, 212–227 (2022).

38. K. Hématy, P.-E. Sado, A. Van Tuinen, S. Rochange, T. Desnos, S. Balzergue, S. Pelletier, J.-P. Renou, H. Höfte, A receptor-like kinase mediates the response of Arabidopsis cells to the inhibition of cellulose synthesis. *Curr. Biol.* **17**, 922–931 (2007).

39. J. Du, A. Kirui, S. Huang, L. Wang, W. J. Barnes, S. N. Kiemle, Y. Zheng, Y. Rui, M. Ruan, S. Qi, S. H. Kim, T. Wang, D. J. Cosgrove, C. T. Anderson, C. Xiao, Mutations in the pectin methyltransferase QUASIMODO2 influence cellulose biosynthesis and wall integrity in Arabidopsis. *Plant Cell* **32**, 3576–3597 (2020).

40. I. Pavelescu, J. Vilarrasa-Blasi, A. Planas-Riverola, M.-P. González-García, A. I. Caño-Delgado, M. Ibañes, A sizer model for cell differentiation in *Arabidopsis thaliana* root growth. *Mol. Syst. Biol.* **14**, e7687 (2018).

41. C. Grierson, E. Nielsen, T. Ketelaarc, J. Schiefelbein, Root hairs. *Arabidopsis Book* **12**, e0172 (2014).

42. K. Vissenberg, N. Claeijs, D. Balcerowicz, S. Schoenaers, Hormonal regulation of root hair growth and responses to the environment in Arabidopsis. *J. Exp. Bot.* **71**, 2412–2427 (2020).

43. M. Samalova, A. Melnikava, K. Elsayad, A. Peaucelle, E. Gahurova, J. Gumulec, I. Spyroglou, E. V. Zemlyanskaya, E. V. Ubogoeva, D. Balkova, M. Demko, N. Blavet, P. Alexiou, V. Benes, G. Mouille, J. Hejatko, Hormone-regulated expansins: Expression, localization, and cell wall biomechanics in Arabidopsis root growth. *Plant Physiol.* **194**, 209–228 (2023).

44. R. Schlüßler, S. Möllmert, S. Abuhattum, G. Cojoc, P. Müller, K. Kim, C. Möckel, C. Zimmermann, J. Czarske, J. Guck, Mechanical mapping of spinal cord growth and repair in living zebrafish larvae by Brillouin imaging. *Biophys. J.* **115**, 911–923 (2018).

45. K. Elsayad, S. Werner, M. Gallemi, J. Kong, E. R. Sanchez Guajardo, L. Zhang, Y. Jaillais, T. Greb, Y. Belkhadir, Mapping the subcellular mechanical properties of live cells in tissues with fluorescence emission-Brillouin imaging. *Sci. Signal.* **9**, rs5 (2016).

46. D. Pereira, T. Alline, S. Schoenaers, A. Asnacios, In vivo measurement of the Young's modulus of the cell wall of single root hairs. *Cell Surf.* **9**, 100104 (2023).

47. J. S. Griffiths, K. Šola, R. Kushwaha, P. Lam, M. Tateno, R. Young, C. Voiniciuc, G. Dean, S. D. Mansfield, S. DeBolt, G. W. Haughn, Unidirectional movement of cellulose synthase complexes in Arabidopsis seed coat epidermal cells deposit cellulose involved in mucilage extrusion, adherence, and ray formation. *Plant Physiol.* **168**, 502–520 (2015).

48. G. Bethke, R. E. Grundman, S. Sreekanta, W. Truman, F. Katagiri, J. Glazebrook, Arabidopsis *PECTIN METHYLESTERASEs* contribute to immunity against *Pseudomonas syringae*. *Plant Physiol.* **164**, 1093–1107 (2014).

49. H.-W. Shih, N. D. Miller, C. Dai, E. P. Spalding, G. B. Monshausen, The receptor-like kinase FERONIA is required for mechanical signal transduction in Arabidopsis seedlings. *Curr. Biol.* **24**, 1887–1892 (2014).

50. A. Malivert, O. Hamant, Why is FERONIA pleiotropic? *Nat. Plants* **9**, 1018–1025 (2023).

51. L. Bacete, J. Schulz, T. Engelsdorf, Z. Bartosova, L. Vaahtera, G. Yan, J. M. Gerhold, T. Tichá, C. Øvstebø, N. Gigli-Bisceglia, S. Johannessen-Starheim, J. Margueritat, H. Kollist, T. Dehoux, S. A. M. McAdam, T. Hamann, THESEUS1 modulates cell wall stiffness and abscisic acid production in *Arabidopsis thaliana*. *Proc. Natl. Acad. Sci. U.S.A.* **119**, e2119258119 (2022).

52. J. Lamers, Y. Zhang, E. Van Zelm, C. K. Leong, A. J. Meyer, T. De Zeeuw, F. Verstappen, M. Veen, A. O. Deolu-Ajayi, C. M. M. Gommers, C. Testerink, Abscisic acid signaling gates salt-induced responses of plant roots. *Proc. Natl. Acad. Sci. U.S.A.* **122**, e2406373122 (2025).

53. A. Petrova, M. Ageeva, L. Kozlova, Root growth of monocotyledons and dicotyledons is limited by different tissues. *Plant J.* **116**, 1462–1476 (2023).

54. S. Mielke, M. Zimmer, M. K. Meena, R. Dreos, H. Stellmach, B. Hause, C. Voiniciuc, D. Gasperini, Jasmonate biosynthesis arising from altered cell walls is prompted by turgor-driven mechanical compression. *Sci. Adv.* **7**, eabf0356 (2021).

55. S. Chen, I. Burda, P. Jani, B. Pendrak, M. N. Silberstein, A. H. K. Roeder, Fibrous network nature of plant cell walls enables tunable mechanics for development. *Nat. Commun.* **16**, 7565 (2025).

56. M. Lan, Y. Zhu, A. Peaucelle, X. Zhu, Y. Liu, X. Cao, A. Gurzadyan, G. Gao, W. Cai, J. Gruel, K. T. Haas, H. Jönsson, O. Hamant, R. Wightman, E. Meyerowitz, W. Yang, CSLD5-mediated cell wall remodelling regulates tissue mechanics and shoot meristem growth. *Nat. Commun.* **16**, 7229 (2025).

57. L. A. Baez, T. Tichá, T. Hamann, Cell wall integrity regulation across plant species. *Plant Mol. Biol.* **109**, 483–504 (2022).

58. A. Chaudhary, Y.-C. Hsiao, F.-L. Jessica Yeh, M. Župunski, H. Zhang, Y. Aizezi, A. Malkovskiy, G. Grossmann, H.-M. Wu, A. Y. Cheung, S.-L. Xu, Z.-Y. Wang, FERONIA signaling maintains cell wall integrity during brassinosteroid-induced cell expansion in Arabidopsis. *Mol. Plant* **18**, 603–618 (2025).

59. L. Vaahtera, J. Schulz, T. Hamann, Cell wall integrity maintenance during plant development and interaction with the environment. *Nat. Plants* **5**, 924–932 (2019).

60. P. Bouvet, C. Bevilacqua, Y. Ambekar, G. Antonacci, J. Au, S. Caponi, S. Chagnon-Lessard, J. Czarske, T. Dehoux, D. Fioretto, Y. Fu, J. Guck, T. Hamann, D. Heinemann, T. Jähnke, H. Jean-Ruel, I. Kabakova, K. Koski, N. Koukourakis, D. Krause, S. La Cavera, T. Landes, J.

Li, H. Mahmodi, J. Margueritat, M. Mattarelli, M. Monaghan, D. R. Overby, F. Perez-Cota, E. Pontecorvo, R. Prevedel, G. Ruocco, J. Sandercock, G. Scarcelli, F. Scarponi, C. Testi, P. Török, L. Vovard, W. J. Weninger, V. Yakovlev, S.-H. Yun, J. Zhang, F. Palombo, A. Bilenca, K. Elsayad, Consensus statement on Brillouin light scattering microscopy of biological materials. *Nat. Photonics* **19**, 681–691 (2025).

61. P. Thévenaz, M. Unser, User-friendly semiautomated assembly of accurate image mosaics in microscopy. *Microsc. Res. Tech.* **70**, 135–146 (2007).

62. C. A. Schneider, W. S. Rasband, K. W. Eliceiri, NIH Image to ImageJ: 25 years of image analysis. *Nat. Methods* **9**, 671–675 (2012).

63. J. Zhang, G. Scarcelli, Mapping mechanical properties of biological materials via an add-on Brillouin module to confocal microscopes. *Nat. Protoc.* **16**, 1251–1275 (2021).

64. E. Edrei, M. C. Gather, G. Scarcelli, Integration of spectral coronagraphy within VIPA-based spectrometers for high extinction Brillouin imaging. *Opt. Express* **25**, 6895 (2017).

65. S. Caponi, D. Fioretto, M. Mattarelli, On the actual spatial resolution of Brillouin imaging. *Opt. Lett.* **45**, 1063 (2020).

66. R. Barer, S. Joseph, Refractometry of living cells part I. basic principles. *Q. J. Microsc. Sci.* **95**, 399–423 (1954).

67. M. González-Guzmán, N. Apostolova, J. M. Bellés, J. M. Barrero, P. Piqueras, M. R. Ponce, J. L. Micol, R. Serrano, P. L. Rodríguez, The short-chain alcohol dehydrogenase ABA2 catalyzes the conversion of xanthoxin to abscisic aldehyde. *Plant Cell* **14**, 1833–1846 (2002).
